# Robotic systems in internet of things: addressing security challenges through threat modeling and penetration testing

**DOI:** 10.1038/s41598-026-48287-8

**Published:** 2026-04-20

**Authors:** Muhammed Rafeeq War, Aqsa Sayeed, Shahid Ahmad Wani, Ayoob Lone, Mohammad Shuaib Mir, Yonis Gulzar

**Affiliations:** 1 Indian Institute of Technology Ropar, Punjab, 14001 India; 2 Central University of Jammu, Jammu, 181143 India; 3 School of Engineering and Technology, K. R. Mangalam University, Sohna Road Gurugram, Haryana, 122103 India; 4 Department of Clinical Neurosciences, College of Medicine, King Faisal University, Al-Ahsa, 31982 Saudi Arabia; 5 Department of Management Information Systems, College of Business Administration, King Faisal University, Al-Ahsa, 31982 Saudi Arabia

**Keywords:** Internet of robotic things (IoRT), Threat modelling, Attack tree, Attack defense tree, IoRT, Penetration testing, Engineering, Mathematics and computing

## Abstract

The increasing prevalence of robotic systems across diverse domains has undoubtedly delivered numerous advantages. However, this proliferation has also exposed these systems to potential security threats, with the ability to cause significant human and financial losses. In this study we propose a proactive approach to risk management using threat modeling techniques. This approach provides a systematic framework for risk assessment, facilitating the identification and prioritization of the most severe threats for possible mitigation. Specifically, we provide Attack-Defense Tree (ADT) based methodology, an extension of the conventional tree formalism which incorporates mitigation nodes representing applicable counter measures at different hierarchical levels. This enhancement enables a comprehensive and structured analysis of security risks. The proposed ADT-based approach is tailored to reinforce the security of the camera component within robotic systems operating on the Internet of Robotic Things (IoRT) environment. Additionally, penetration testing is conducted to empirically evaluate the vulnerability of robotic devices to flooding-based denial-of-service (DoS) attacks. Experimental validation using the AlphaBot platform demonstrates the feasibility of executing a successful DoS attack on its camera module. These findings underscore the urgent need for anticipatory security strategies in IoRT systems and highlight the critical importance of robust, preemptive threat modeling to ensure cyber-resilience in modern robotic infrastructures.

## Introduction

The Internet of Robotic Things (IoRT) technology encompasses robotics, cloud computing, the Internet of Things (IoT), digital twins, virtual reality, and artificial intelligence^1^. IoRT integrates robotic agents with IoT systems to create new possibilities across industrial and scientific domains. It enables robots to send and receive data to and from other devices and people^2^. IoRT provides a robust framework for connecting devices to facilitate machine-to-machine (M2M) and machine-to-human (M2H) data exchange using standard protocols such as TCP/IP. Various networks—including 3G, 4G, LAN, Bluetooth, RFID, and NFC—are employed to transfer data. Devices such as routers, controllers, and gateways are used to enable connectivity^3^. Cellular connectivity and short-range communication technologies (e.g., BLE, Wi-Fi, 6LoWPAN, Near Field Communication (NFC), and Broadband Global Area Network (BGAN)) facilitate seamless connections among nearby robotic devices. For long-distance communication robotic systems utilize medium-range technologies such as ZigBee, Z-Wave, Low Power Wide Area Network (LoRaWAN), and Worldwide Interoperability for Microwave Access (WiMAX). Currently, a range of widely adopted internet communication protocols include CoAP, MQTT, IPv6, XMPP, DTLS, UDP, AMQP, DDS, and LLAP. The main functionalities of these protocols are summarized in Table 1^4–6^.

The IoRT has a wide-ranging impact on human living standards. Preprogrammed robots have helped industries to enhance productivity by providing unheard-of levels of accuracy and 24 × 7 operational availability. Several firms employ robots for complex, pivotal, and tough tasks such as product assembly, welding, product testing, quality control, packing, and so on. The IoRT’s physical operation classes encompass subterranean and ground, planetary exploration and space, undersea and marine, and hybrid site operations, as well as planetary exploration and aerial operations. Each class has a unique set of powers^7^. Security has been a fundamental challenge for the interconnectivity of robotic systems^8^. IoRT faces serious problems regarding protection and security while enabling essential association among sensors, networks, and robotics. Secure communication methods for data transmission and processing are continually in demand, such as encrypted transmission between robotic entities. Secure frameworks in terms of integrity and confidentiality are required for data transfer among robotic things. The IoRT system must be supported by physical access to security measures for data verification, trust & privacy, and data confidentiality^9–12^. The dependability of IoRT systems is enhanced by hardening end-to-end security, digital identities, and mobile data services. This progression is driven by advancements in robotic cognition facilitated by modern AI algorithms^7^. A system is usually vulnerable to enormous attacks; to overcome these system threats, various methodologies are used to test the system’s security before communication^13^. Cyber-attacks of many forms have increased the security hazards to modern computer and networking systems. These hazards represent significant security concerns for individuals, society, and businesses^8^.

In this study, we focus on threat modeling through penetration testing to evaluate the system’s ability to detect and analyze vulnerabilities. Utilizing the Metasploit framework, we conduct a series of controlled attacks to examine the robustness of robotic systems, with a particular focus on the security of robotic camera modules—critical components in many IoRT applications. While various types of attacks may target essential robotic functionalities, this work specifically.

**Table 1:** Functions of communication protocols.

**Fig. 1:**
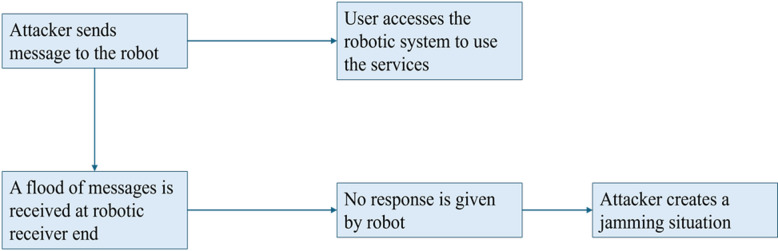
DoS attack performed by the attacker.

Multicast support Publish/subscribe messaging Packet-switched networking Real-time instant messaging Spreading of network embedded systems as a substitute to TCP Message queuing for a middleware environment Providing privacy to datagram protocol Directly addressing publish/subscribe-based communication for real-time and embedded systems, and Lightweight local automation.	

emphasizes vulnerabilities associated with robotic vision systems. To systematically represent these threats, we propose a novel Attack-Defense Tree (ADT) that captures potential attack vectors and corresponding mitigation strategies for robotic cameras. As proof of concept, a denial-of-service (DoS) attack derived from the proposed ADT is executed on a robotic platform. As illustrated in Fig. 1, the attack involves flooding the robot with an excessive number of messages, ultimately rendering the system unresponsive to legitimate user commands. This induced jamming condition highlights a critical security concern and underscores the necessity of preemptive threat modeling in securing robotic components.

The primary contributions of this work are as follows:

1. **A unified proactive-reactive security framework**: We synthesize isolated threat-modeling methodologies (STRIDE, DREAD, and PASTA) into a cohesive framework, moving beyond theoretical analysis to establish a comprehensive security lifecycle for the Internet of Robotic Things (IoRT).
2. **Formulation of an IoRT-specific attack-defense tree (ADT)**: We introduce a novel taxonomy and quantifiable ADT specifically tailored for robotic edge devices. This provides a clear, structural mapping of both attack vectors and their corresponding mitigation strategies in resource-constrained environments.
3. **Empirical validation on physical edge robotics**: Diverging from contemporary studies that rely on simulation or generalized IT architectures, we validate our theoretical threat models through active physical penetration testing. By executing targeted reconnaissance and SYN flooding DoS attacks on an AlphaBot testbed, we demonstrate the real-world exploitability of robotic systems.
4. **Actionable mitigation methodology**: We bridge the gap between abstract risk assessment and practical defence by directly integrating the empirical results of our penetration testing back into the ADT. This establishes a reproducible research methodology for continuous vulnerability identification and mitigation in IoRT systems.

The organization of the remaining part of this paper is arranged as follows: Sect.  2 presents the related literature and Sect.  3 discusses the threat modeling concepts. Penetration testing for robotic devices is proposed in Sect.  4. Section  5 is based on the results and discussion. Finally, Sect.  5 presents conclusion, limitations along with the future scopes.

## Literature survey for the study

The IoT network layer is vulnerable to several attacks, such as RPL attacks and 6LoWPAN attacks. Furthermore, IPv6 attacks present significant risks, including fragmentation, authentication, confidentiality, replay, and spoofing attacks. Similarly, RPL attacks include RPL-specific attacks, such as rank attacks, version attacks as well as WSN-inherited attacks including black hole attacks, sinkhole attacks, and wormhole attacks. Sayeed et al.^11^ proposed a review on IoT secure routing and security in network layers. The authors reported that to overcome network attacks, trust-based models are focused on enhancing the security of the system. The trust properties of models are asymmetrical, partially transitive, and context sensitive. The authors also mentioned the trust metrics including QoS trust metrics, communication trust, and mobility. Bradbury et al.^14^ proposed a trust model-driven system architecture for IoT devices. The IoT system architecture has a finite lifespan but can use batteries; at that time, firmware updates are required. The multiple applications running on this IoT system are mutually trusted. Threats can be classified into two categories as follows (i–ii):

(i) CIA stands for “Confidentiality, Integrity, and Availability”.
(ii) STRIDE stands for “Spoofing, Tampering, Repudiation, Information Disclosure, DoS, Escalation of Privilege.

Threat modeling methods like the Cyber Kill Chain and Process for Attack Simulation and Threat Analysis (PASTA) are used to mitigate the above-mentioned threats. Ankele et al.^15^ discuss threat modeling, penetration testing, and security analysis in IoT and Industrial Internet of Things (IIoT) devices. Cyber-physical systems are the main technology that acts as a bridge between the IoT and IIoT systems. To make IoT/IIoT devices secure and safe, the system needs to be focused on the design, implementation, and verification of the Security Development Lifecycle (SDL) process. The security analysis, threat modeling, and penetration testing address the design, threat part of the model, and verification of the fulfillment of security goals, respectively. Various attacks are possible on different components of the Internet of Robotic Things (IoRT), which integrates interconnected robots with internet connectivity, enhancing functionality and communication. Understanding its network complexity is critical for preemptively identifying and addressing security vulnerabilities, such as sensors being susceptible to DoS and spoofing attacks, whereas actuators are vulnerable to authentication failures. Storage modifications, data leakage, and calculation errors occur in IoT/IoRT/IIoT technology.

Table 2 presents components of the IoT and their corresponding possible attacks. Rostami et al.^16^ present threat models and metrics for hardware security to overcome hardware Trojans. Using threat models, some defenses are acquired to tackle hardware attacks. Some important metrics mentioned by the author to overcome the attacks are obfuscation, watermarking, fingerprinting, and metering^17^. Luonis et al.‘s^18^ presents a thorough examination of attack defense mechanisms, particularly focusing on the Wi-Fi fabrication attack within IoT wireless technologies. They provide valuable insights into potential threats and offer effective strategies for mitigation, thereby bolstering security across IoT environments. Fei et al.^19^ have proposed an attack defense tree for advanced persistent threats (APT). In recent years, APT attacks have become a means of compromising national defense, social harmony, and business expansion. APT attacks are recognized by a simple objective, a prolonged incubation time, and various means. APT attacks and protection are classified into four types^20^ as follows (a–d): (a) big data analysis, (b) social engineering assessment, (c) game-theoretic evaluation, and (d) visualization techniques analysis. An attack defense tree can be created using situational awareness data from the small and medium Internet, which is obtained from the data flow gathered by the probe placed on the corporate route. It can also be constructed with the assistance of target awareness from online data flow gathered by the investigation operated on the organization path. Attacks usually include (a–e): (a) SQL injection to the host of the external network, (b) Trojan email, (c) rainbow table attacks and background password cracking (d) malicious file upload and (e) unauthorized access via social workers. Maciel et al.^21^ developed hierarchical models to elucidate the functionality of essential system components and evaluate the effects of DDoS attacks on system availability. Their methodology involved examining attack tree indices to gauge how simultaneous threats impact computer systems, identifying scenarios that result in significant system downtime. The results provide valuable insights into vulnerabilities and strategies for mitigating DDoS attacks. However, the complexity of modeling various attack scenarios and the dynamic nature of cyber threats may pose challenges to the accuracy of the study’s findings. Kalugina et al.^22^ introduced a method utilizing software modeling strategies to enhance information security risk assessment. Their approach improves threat evaluation by swiftly compiling an attack tree from up-to-date company resources. While offering a more efficient assessment of security risks, challenges may arise from the necessity of maintaining current information and implementing intricate software modeling techniques. The initial phase of the info-system security and the probabilistic properties of an invader determine the info-security threats. An approach for determining the probability of threat deployment based on the fuzzy logic algorithm is presented to simplify the procedure of designing threats to information security. The author makes use of Russia’s threat.

**Table 2:** Major security attacks on the components of robotic things.

Components	Attacks
Sensors	Spoofing, Denial-of-Service attacks.
Actuators	Authentication failure, relay attacks, deadline miss.
Storage	Storage modification, data remanence.
Computing	Info leak, timing fault, computing fault.
Communication	Snooping, man-in-the-middle, packet modification, resource stalling.

database, FSTEC. Offline updates are performed on the software database. Morikawa et al.^23^ proposed threat Offline updates are performed on the software database. Morikawa et al.^23^ proposed threat tree templates for constructing the attack tree–attack defense tree for threat modeling. In order to estimate risk and plan for mitigation, the trees are used to recognize how and under what conditions dangers can be realized. Moreover, it is challenging for a researcher to create appropriate trees because security expertise is needed to identify possible attacks. Threat Tree Templates (TTT) offer structured frameworks to analyze threats and vulnerabilities in systems or networks. These frameworks delineate potential attack scenarios, assisting researchers in methodically assessing risks and formulating mitigation strategies. Through hierarchical organization of threat data, TTT fosters a deeper comprehension of security risks, thereby facilitating the creation of robust defense mechanisms. TTTs are mentioned to assist non-expert observers in designing threat trees. Templates are prototypes of threat trees; branches depict attack possibilities, security breaches, and mitigation strategies. The template keyword system seeks to eliminate superfluous circumstances. A detailed summary of various threat modeling methods along with challenges and observations is discussed in Table 3. This Table presents a survey of existing IoT security threat modelling frameworks, covering ADT, penetration testing, PKI, threat tree templates, and IoTSec UML/SysML extensions. ADT models both attacks and countermeasures in a unified structure, while PKI authenticates identities and secures data exchange. Threat tree templates and UML extensions further support systematic threat identification and standardized IoT security design from early development stages. Despite their contributions, all studies consistently lack AI/ML integration, show limited alignment with modern standards such as NIST or ISO 27,001, and demonstrate restricted scalability, gaps that contemporary hybrid approaches are beginning to address.

Recent literature shows a clear shift toward securing algorithms, privacy, and distributed learning in IoRT. Table 4 presents a comparative analysis of the proposed framework against existing IoT, IoRT, and system security literature. Notable contributions include blockchain-based privacy by Zhai et al.^24^, Informer-based vulnerability testing by Shi et al.^25^, federated defense frameworks by Zhou et al.^26^ and Natarajan^27^, and NIST AI RMF adaptation by Karim et al.^28^. While sophisticated, these works predominantly rely on simulation or cloud-based environments, leaving physical hardware validation largely unaddressed. Foundational works by Khalid^1^, Ray^2^, and Verma et al.^29^ provide valuable architectural overviews of IoRT vulnerabilities but remain largely conceptual. Threat modelling approaches by Bradbury et al.^14^ and Ankele et al.^15^ advance IoT security assurance yet lack integration with reactive defence modelling such as ADT. Similarly, systemic frameworks like Abakumov et al.^30^ and Yoshino et al.^31^ establish strong industrial foundations without quantifiable ADT support.​ The proposed work uniquely bridges these gaps by combining proactive threat modelling (STRIDE, DREAD, PASTA) with reactive ADT, validated through active penetration testing specifically SYN flooding DoS attacks on a physical, resource-constrained AlphaBot edge robotic testbed. This makes it the only reviewed framework offering an end-to-end, empirically validated security assessment for physical IoRT devices.

**Table 3:** Comparative analysis of IoT security threat modeling frameworks.

Author	Domain	Method	Brief description	AI/ML integration	Standardization alignment	Scalability	Limitation	Key outcome
Sayeed et al.^11^	Protected IoT routing	Trust based methods	Trust based countermeasures mapped to specific IoT routing threats.	None	Partial	Low	Resource constraints, mobility, routing attacks	Establishes trust criteria and identifies research gaps.
Bradbury et al.^14^	Resource constrained IoT	Threat modeling, PKI, attack trees	Models threats in trust-based task offloading through actors, components, and attack paths.	None	Partial (PKI)	Moderate	Trust models identified but not implemented	Precise ADT for trust offloading architecture produced.
Ankele et al.^15^	IoT and IIoT devices	Threat modeling, penetration testing	Evaluates penetration testing and threat modeling tools for IoT and IIoT devices.	None	Partial	Low	Tests exhaust quickly, limiting analysis depth	Defines essential input and metadata parameters for IoT security testing.
Louniset al.^18^	Wireless IoT technologies	Countermeasures for wireless attacks	Discusses wireless threats such as spoofing, replay and Bluetooth attacks with countermeasures.	None	None	Low	Focuses mainly on short range technologies	Provides an ADT for each wireless attack.
Feiet al.^19^	APT attack modeling	Attack Defense Tree (ADT)	Uses AHP to assign weights and probabilities to attack and defense nodes.	Partial	None	Moderate	Expert system reasoning needs improvement	Defense node reduces attack probability from 0.714 to 0.614.
Maciel et al.^21^	DDoS attack modeling	Attack threat modeling	Uses SecurITree to model DDoS attacks and analyze defense strategies.	None	None	Moderate	Time series mitigation not addressed	Attack probability observed as 95.8% amplification, 82% TCP SYN, 71% hping3.
Kaluginaet al.^22^	Information system security	Attack tree method	Automated tool evaluates vulnerabilities using encryption, ACLs, IPSec and SSL protections.	None	Partial	Low	Limited to small and medium enterprises	Calculates threat levels for selected asset vulnerabilities.
Morikawaet al.^23^	Threat tree modeling	Threat tree templates	Reusable templates with keyword filtering for systematic threat identification.	None	None	Moderate	Templates may become complex and time consuming	Enhances threat identification quality and coverage.
Robles-Ramirezet al.^32^	IoT security modeling	IoTSec UML and SysML extension	UML and SysML extensions support visual security modeling throughout IoT lifecycle.	None	Partial	Moderate	Requires more real world validation	Allows addition of new diagrams and constraints for IoT security.
Sion et al.^33^	Information threat modeling	Risk assessment model	Integrates and decomposes privacy risks for system wide risk evaluation.	None	Partial	Low	High computational complexity	Calculates risk factors, retention periods and threat event frequency.

**Table 4:** Comparative analysis of IoRT security approaches.

References	Target domain	Core security approach	Threat modeling methodology	Attack/defense trees utilized?	Physical hardware testbed	Primary attack vectors evaluated
Khalid^1^& Ray^2^	IoRT Architecture	Literature Review & Conceptual	None	No	None (Survey)	General theoretical vulnerabilities
Bradbury et al.^14^	Resource-constrained IoT	Trust-based Task Offloading	General Threat Modeling	No	Simulation	Node compromise, trust manipulation
Ankele et al.^15^	IoT / Industrial IoT	Automated Security Assurance	STRIDE / General	No	General IoT components	Broad IoT network threats
Fei et al.^19^& Maciel^21^	Enterprise / Computer Systems	Quantifiable Modeling / Impact	None	Yes (ADT/Attack Tree)	Simulation/Theoretical	APTs, DDoS
Das et al.^32^	IoRT Surgical Digital Twins	Digital Twin Integration	None	No	Conceptual	Healthcare cyber threats
Wang et al.^33^	IoRT Sensing & Communication	Adaptive ISCC Sensing	None	No	Simulation	None (Performance focus)
Zhai et al.^24^	IoRT EV Charging Networks	Privacy-preserving Blockchain	None	No	Simulation	Identity & Privacy leakage
Abakumov^34^& Yoshino^31^	Industrial / RT-Middleware	IMECA / Protocol Optimization	IMECA	No	Industrial / RT-Components	General Cyberattacks, Latency
Abs Yaacoub^30^	Modular IoT Robotics	LCAPBB Auth Protocol	None	No	Blinky Blocks (Modular)	Cyber-physical / Comm. threats
Shi et al.^25^	IoRT Applications	Informer-based App Testing	None	No	App Testing Framework	App sequence crashes / Bugs
Karim et al.^28^	IoRT (LLM Workloads)	NIST AI Risk Management	Risk Management Framework	No	Conceptual / Simulation	Adversarial attacks on AI
Natarajan^27^& Zhou^26^	Cloud/Federated IoRT Models	Federated Defense / SecFFT	None	No	Cloud / Network Simulation	Model Poisoning, Backdoor Attacks
Verma et al.^29^	Robotic Systems Cybersecurity	Systematic Review Framework	None	No	None (Survey)	Broad vulnerabilities
Proposed Work	IoRT (Edge Robotics)	Hybrid (Proactive + Reactive)	STRIDE, DREAD, PASTA	Yes (ADT)	Yes (Physical AlphaBot)	Reconnaissance, DoS (SYN Flooding)

## Threat modelling

It is a method of security analysis to aid in product security. The technique is advocated as a part of the Microsoft Secure Development Lifecycle. Threat modelling examines a system from the perspective of a potential attacker rather than a defender. The threat model first breaks the application. After the threat is determined, its rank is determined. The final step is threat mitigation and countermeasures. The resulting report is the application’s threat model^9,16,35,36^. Threat modelling is a step-by-step procedure of system security. The various threat modeling process steps are as follows (i–iv):

(i) **Decompose the application**: The target system is decomposed into components, preferably through Data Flow Diagrams (DFDs) that focus on the creation, transfer, and storage of data. The DFD consists of four entities: processes, external entities, data stores, and data flows. This entails developing use cases to better comprehend how an application is applied. The decomposition helps in the following:

- Recognizing access to determine how a potential attacker might interact with the system.
- Labeling assets that would be of interest to the attacker.
- Defining trust levels to portray the access privileges that software will confer to outside entities.

**Fig. 2:**
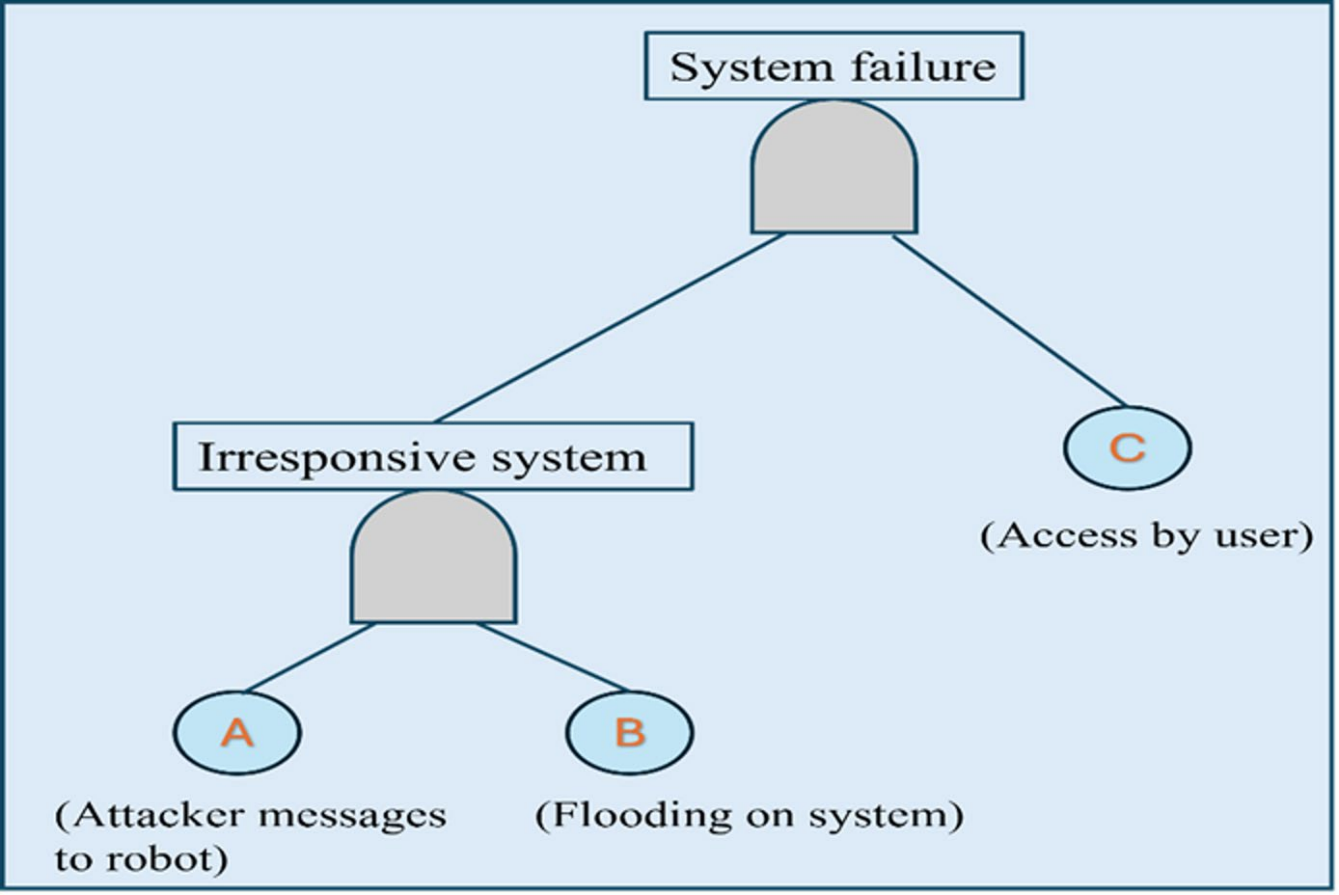
Attack tree.

In order to generate DFDs, this information is kept in a document for the threat model. The DFDs depict the various paths through the system, emphasizing the authorization limits.

(ii) **Threat identification and categorization**: Possible threats for a system can be classified using a threat classification tool (e.g., STRIDE or the Application Security Frame (ASF)). The ASF describes threat groupings such as authorization, authentication, configuration management, data validation, database data protection, data in transit, auditing and logging, and exception management. The purpose of threat classification is to assist in recognizing threats from both the attacker and defensive sides. DFDs assist in identifying probable threat victims from the assailant’s viewpoint, such as data sources, processes, data streams, and user interfaces. A threat classification tool (e.g., STRIDE) can assist in determining threats by defining attacker aims including spoofing, tampering, repudiation, information disclosure, denial of service, and elevation of privilege^37,38^. Table 5 contains a list of basic threats labeled using STRIDE, as well as their security measures. In spoofing, an attacker gains access to the important credentials of another user. To overcome this situation, system authentication is done. An attacker tampers with the data during data transmission. To overcome tampering, data integrity is needed. Non-repudiation, confidentiality, availability, and authorization are the primary security services that can prevent possible attacks.
(iii) **Threat analysis and ranking**: The risk is defined as the possibility for an asset to be damaged, lost, or destroyed as a result of a threat exploiting system vulnerabilities. It is vital to discover and characterize any flaws that might lead to an exploit. An analyst can use Damage, Reproducibility, Exploitability, Affected Users, and Discoverability (DREAD) or other methodologies to predict the quantity of risk, the weaknesses of organizational structures, the occurrence of threats, and the cost of the ris^36,39^. Qualitative evaluation approaches entail locating, classifying, and rating undesirable instances based on the ability of the assessor, awareness, tactics, and unusual situations in information management. Quantitative assessment methodologies examine an organization’s risk level and generate numerical indicators using mathematical calculus, statistics, and probability concepts. Basic quantitative evaluation approaches include statistical parameter analysis, Markov analysis, and Bayesian network modeling. Qualitative analytical risk evaluation with subjectivity restricts the model to a unified system^40^. A quantifiable risk evaluation method such as CORAS is a model-based method of analyzing security risks. Risk is computed using the qualitative risk model as (Threat Occurrence Probability) × (Cost to the Organization)^41^. Various risk factors can be employed to categorize and rank the threats as Low, Medium, or High risk. A DREAD score is determined by a point system of numbers 1–10, depicting low to high magnitude, to help threat comparisons^42^. All of these parameters, in turn, describe the attack severity, attack replicating level, attack duration, percentage of affected users, and threat detection by the attacker. To evaluate a threat ranking^22^, the threat expert responds to questions for every risk factor, as shown in Table 6. Threat modeling is a systematic and tactical method for detecting and listing threats to an application system in order to reduce risk and potential implications. A threat tree can help prove how and under what conditions the threat could be realized. Figure 2 illustrates a corresponding attack tree for the DoS attack indicated in Fig. 1, where A represents attacker messages on the robot AlphaBot and B indicates flooding of the system. The system becomes inaccessible because of both of these circumstances, C stands for user access. When the system is inaccessible and a user tries to access it, the system fails. To fulfill the criteria, two AND gates are utilized, such that the A AND B operation results in an unresponsive system, and the first AND operation result is ANDed with C, resulting in system failure.
(iv) **Countermeasures and mitigation**: Sort the threats by priority and then consider mitigations and countermeasures against them if needed. Provided below (iva–ivf) is a summary of threats and their countermeasures^38,43^:

- **Authentication**: Authentication and credential tokens are encrypted while in storage and transit to combat threats to authentication. The use of brute force, dictionary, and replay attacks should not be possible on protocols. Therefore, strict password rules are implemented. SQL authentication is substituted with trusted server authentication. Passwords are kept using salted hashes, and legitimate usernames and password hints are not revealed via password resets. Denial-of-service attacks do not occur from account lockouts^18^.
- **Authorization**: Powerful ACLs are applied to enforce allowed access to resources in order to protect against threats to authorization. To limit access to particular operations, role-based access restrictions are utilized. When it comes to user and service accounts, the system adheres to the least privilege principle. The presentation, business, and data access layers are properly set for privilege separation^44^.
- **Data protection in storage and transit**: Standard encryption algorithms and the appropriate key sizes have been applied to mitigate the dangers. To safeguard data integrity, hashed message authentication codes are utilized. Secrets are cryptographically safeguarded throughout storage and transportation, including keys and secret data. Keys are safeguarded in built-in secure storage. No sensitive information or login credentials are transmitted over the wire in clear text^45^.
- **Data validation / parameter validation**: format, Data type, range checks, and length are mandated in order to mitigate the dangers. The client validates all of the data it sends. No security decision is dependent on variables that can be changed, like URL parameters. Use of input filtering enables list validation. Encoding for output is employed^46^.
- **User and session management**: Unimportant information is saved in the cookie in clear text as a precaution against threats. The authentication cookies’ data is encrypted. Cookies have an expiration date set. Replay attacks are not successful on sessions. Cookies used for authentication are protected via secure communication channels. When carrying out crucial tasks, the user must re-authenticate. At logout, sessions expire^47^.
- **Auditing and logging**: The sensitive information (such passwords and PII) is not logged as a precaution against attackers. To prevent unwanted access, log files are enforced with access controls (such ACLs). In order to achieve non-repudiation, integrity rules (such as signatures) are enforced on log files. Log files offer an audit trail for private processes and crucial event logging. On numerous servers, auditing and logging are enabled throughout the tiers^18^.

**Table 5:** Threat classification technique^34^.

STRIDE	Threat summary	Security Services
Spoofing	Threat activity aims at gaining access to and using the credentials of another user, such as password and login.	Authentication
Tampering	Threat activity intended to deliberately modify or alter tenacious data, such as db entries, and data modification in transmission amid two computers via an open channel-Internet.	Integrity
Repudiation	Threat exploit is intended at conducting illegal activities on a platform that is incapable of tracing the activities.	Non-Repudiation
Information disclosure	Threat activity expecting to view a file to that one was not given entree or to steal transmitted data.	Confidentiality
Denial of service	Threat operations try to restrict entree to legitimate users, such as creation a web service inaccessible or ineffective for some time.	Availability
Elevation of privilege	Threat operation aims to gain unrestricted entree to assets to obtain unapproved entree to data or damage a organization.	Authorization

**Table 6:** Threat rank analysis (DREAD)^22^.

Meaning	Role
Damage	Magnitude of damage if the attack is succeeful?
Reproducibility	How simple is it to replicate an attack?
Exploitability	How much duration, effort, knowledge, and experience are required to manipulate the threat.
Affected Users	If a threat is manipulated, what portion of users will be impacted?
Discoverability	How simple is it for an intruder to explore this threat?

Determine how an attacker would be able to compromise a system using threat modelling in IoRT, and then make sure the right mitigations are in place. Threat modelling compels the design team to think about mitigations while developing the system rather than after it has been put into place. An IoRT architecture typically has multiple components and zones that are divided up as part of the threat modelling process. Device, field gateway, cloud gateways, and services are some of the components. Components are a general method of system segmentation; each zone usually has unique requirements for data, authentication, and authorisation. Zones can also be used to limit the impact of low trust zones on higher trust zones and to isolate harm^48^. There are several concerns in robotic things like security, safety, accuracy, and trust about the deployment of robots in critical infrastructures like industrial, medical infrastructures, etc.

Security is primarily concerned with the level of protection afforded to these robots against various types of cyberattacks. In such scenarios, threat models are used to overcome any possible threats in IoRT^16^.

### Threat modeling methods

Commonly used methods for threat modeling include attack trees, ADTs, penetration testing methods, PKI, and the IoT UML extension.

#### Attack tree

A simulation procedure based on attack trees is one approach to creating a threat model. Attack graphs are typically examined as part of a network security evaluation. In general, such an analysis entails sequentially scanning all network devices for known security holes. Attack trees enable researchers to simplify their task of investigating the security and security issues of a developed software system^19^. Attack trees allow you to frame all potential problems for each application entity. The end result is a document that includes a list of discovered vulnerabilities as well as recommendations for their removal. Attack trees are a summary of attack possibilitiesfor achieving a particular goal, which is found at the top of the tree. A good proportion of threats could be described for each software product. A goal, a threat to which an element or connection is vulnerable, or a sub-goal to which threats are conformed is represented by each node in the attack tree. These attack trees are intended to reflect archetypal dangers rather than to be exhaustive^14^. The threat notifications can be accomplished by the following: (i) Assigning a likelihood of occurrence of threats. (ii) Researching the attacker’s actions and calculating how long it will take the intruder to destroy a system, and (iii) The analyst guides the victim to invest in mitigating attacks on its facilities.

**Fig. 3:**
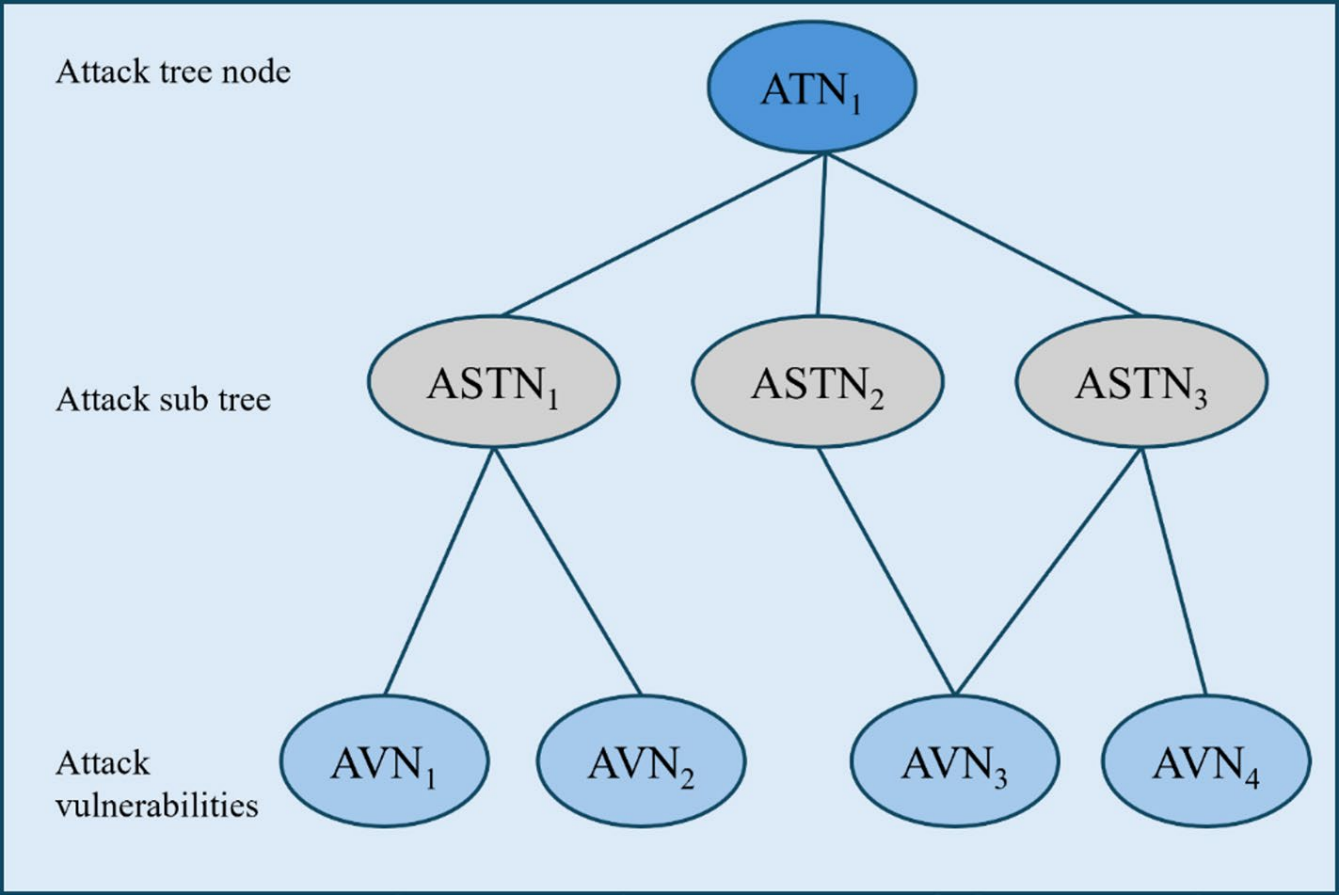
Hierarchical attack tree.

This knowledge enables us to optimize the security built into a model in terms of possible threats. Figure 3 depicts an organized and hierarchical AT illustration. Using a bottom-up methodology, the attack tree’s operations begin at the leaf nodes, where malware actions are performed. The combined impact of the suggested attacks must be designed using the logical operators AND or OR. Every logical operator is subjected to a computation to finally arrive at the attacker’s primary goal^23^. Suppose we have an attack tree AT, with $\:v=\{{v}_{1},{v}_{2},\dots\:\dots\:\dots\:\dots\:,\:{v}_{m}\}$set of attack nodes,$\:e=\{{e}_{1},{e}_{1},\dots\:\dots\:\dots\:.,{e}_{n}\}$edges of the tree (an arc from one node to another), and $\:\mathrm{r}=\{\wedge\:,\vee\:\}$ operator function of AT. Two types of operations AND and OR are possible in the attack tree and attack defense tree. The equation of the attack tree can be given as Eq. (1):

1 $$\:AT=\{v,e,r\}$$

Figure 3 depicts an instance of a threat tree. The root node (ATN1) presents a security risk to the system. Each of the other successor nodes denotes a sub-threat (ASTN1): a situation or event that can occur as a result of the parent. When a node has several child nodes, their connection is by default disjunctive (∨). A conjunctive (∧) relation is present if the corners are denoted as “AND.” Although a threat tree is a crucial tool for analyzing potential risks, constructing its attack paths requires security knowledge, making it the most difficult part of the threat modeling process^23^.

#### Attack defense tree

The goal-oriented perspective provided by attack trees aids in the modeling of multilayer attacks. The attack tree, however, does not take into account defensive measures or actions. These countermeasures can be represented in the attack-defense tree. For an ADT, with$\:\:\mathrm{v}=\{{\mathrm{v}}_{1},{\mathrm{v}}_{2},\dots\:\dots\:\dots\:\dots\:,\:{\mathrm{v}}_{\mathrm{m}}\}$ set of attack nodes, $\:\mathrm{d}=\{{\mathrm{d}}_{1},{\mathrm{d}}_{2},\dots\:\dots\:\dots\:\dots\:,\:{\mathrm{d}}_{\mathrm{p}}\}$set of defense nodes, I intermediate nodes,$\:\mathrm{e}=\{{\mathrm{e}}_{1},{\mathrm{e}}_{1},\dots\:\dots\:\dots\:.,{\mathrm{e}}_{\mathrm{n}}\}$edges of the tree, and $\:\mathrm{r}=\left\{\wedge\:,\vee\:\right\}$operator function, the equation of the ADT is in (2).

2 $$\:\mathrm{A}\mathrm{D}\mathrm{T}=\{\mathrm{v},\mathrm{d},\mathrm{e},\mathrm{r}\}$$

**Fig. 4:**
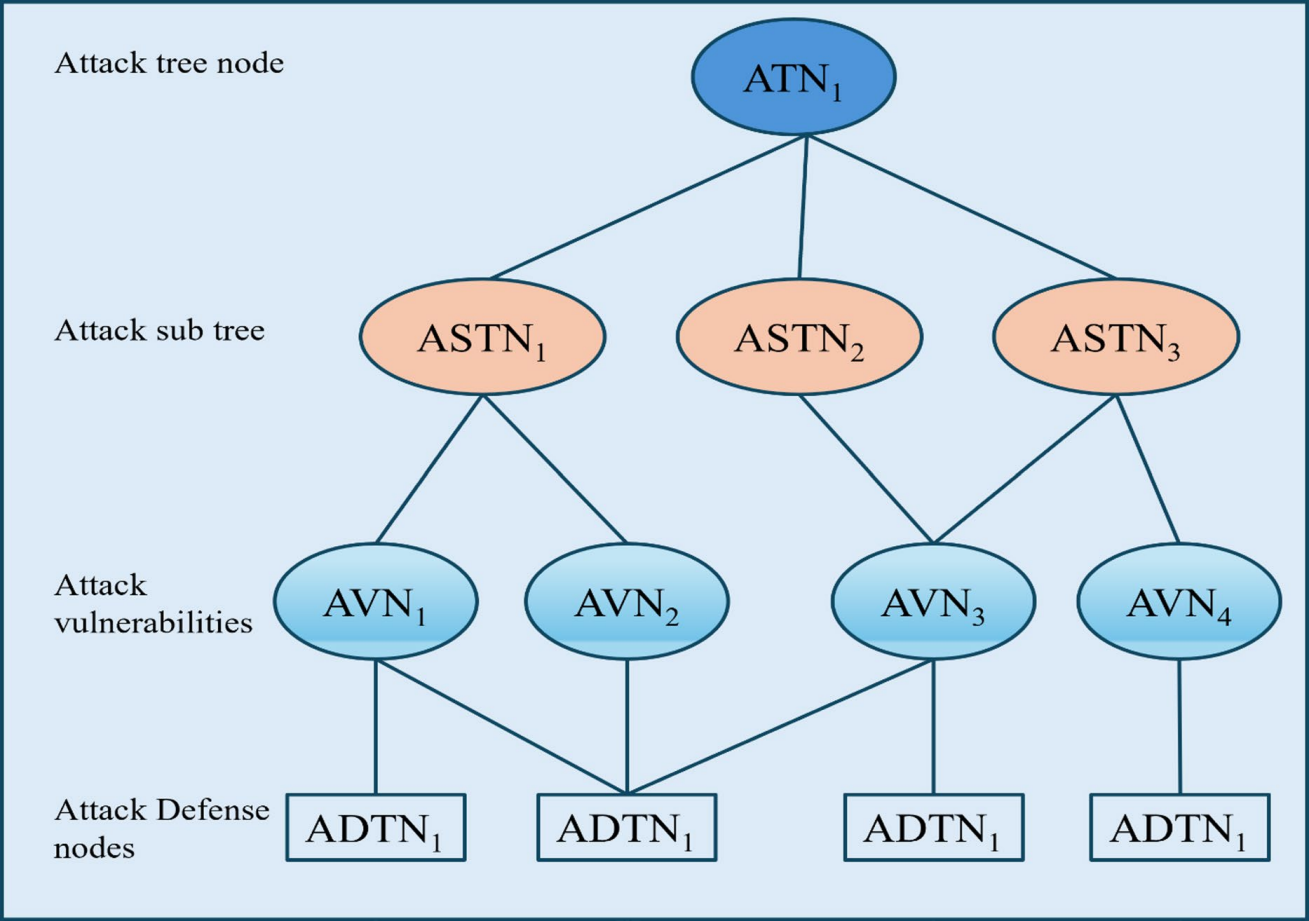
Attack defense tree.

Figure 4 depicts a hierarchical and structured ADT illustration. Attack Defense Trees employ a bottom-up strategy, beginning with the root nodes, where the main attack is mentioned. The attack goal is then identified, and the corresponding countermeasure is applied to the system attack. The method for creating and analyzing the ADT model is given as follows (a–d):

- Methods such as vulnerability scanning and intrusion detection are used to locate the intruder’s attack target. The execution method and attack tree are then established.
- The construction of the ADT requires defensive measures and an attack tree.
- Examining attack nodes and attack leaf node targets, identifying security features and estimating the weight of security features, estimating attack node probability, and establishing relationships.
- Evaluating the defense node and its impact on the attack node.

Several factors influence the occurrence and efficiency of defense leaf nodes and attack leaf nodes. As a result, multiple attributes of leaf nodes are determined. The attributes are converted to utility using attribute-utility theory. Eventually, the probability of attack nodes is evaluated^18,23^. Table 7 represents the various attack-defense attributes in threat modeling from the attacker’s and defender’s points of view. The attack-defense tree is comprised of various threats, attacks, and mitigations, in which the attacker evaluates attack cost, benefit, and difficulty, and the defender evaluates the cost of defense, the benefit of defense, and the difficulty of defense.

**Table 7:** Attack defense tree attributes.

Domain	Attributes
Attacker considers	Attack cost
	Attack benefit
	Difficulty of attack
Protector considers	Cost of defense
	Advantage of defense
	Difficulty of defense

#### Penetration testing

Penetration testing is a method used to test a system, including social engineering, for analyzing the vulnerabilities and threats to the system. These tests may be run from both the inside and outside of the systems under test to ensure that all conceivable attacker options are covered. The purpose of each penetration test is to provide recommendations and ideas for resolving the detected challenges^15,49^. Table 8 discusses the penetration testing attack techniques, phases, tools, and input. Pen-testing includes white-box, grey-box, and black-box testing. Pen-testing is a five-step procedure that begins with reconnaissance and then moves through scanning, gaining access, maintaining access, and covering tracks. To perform a penetration test, different pen-testing tools such as Nmap, Metasploit, SQLMap, Subfinder, Dmitry, and the Burp Suite are necessary. Reconnaissance collects host names, network addresses, IP addresses, and hardware.

interface details. During scanning, port numbers, IP addresses, and operating system information are used as input.

### Related mathematical formulae

**Probability of AND and OR operations: **Probability of AND (p&) and probability of OR (po) operations for the attack tree is given in the Eqs. (3)–(4), respectively.

3 $$\:{p}_{\&}\:=\:\prod\:_{i\:=1}^{n}{L}_{i}$$

**Table 8:** An illustration of penetration testing^15^.

Penetration testing		
Attack procedure	Black-Box testing	The target system’s basic information is accessible.
	Grey-Box testing	The opponent has little information at his disposal.
	White-Box testing	The attacker has access to extensive background details.
Penetration testing phase	Reconnaissance	Information on the specific system, application software, and users is collected to subsequently attack the specific system.
	Scanning	Procedural tools are employed to expand the attacker’s understanding of the specific system.
	Attaining access	The attacker purposefully attempts to attack the methods using the data obtained in earlier phases.
	Maintaining access	An assailant must keep a persistent decryption key to collect the most feasible data.
	Covering tracks	To stay anonymous, an invader must remove any hints, like log files.
Penetration testing tools (PTT)	Nmap	An open-source network scanner is utilized throughout the investigation and scanning phases. It searches single IP addresses as well as IP ranges and offers critical info.
	Metasploit	An open-source structure is used for info collection as well as exploit execution. The utility includes a collection of pre-installed exploits and auxiliary scripts.
	Sqlmap	Free penetration testing tool to streamline and automate SQL database exploits. It may also be used to determine the database utilized. As a reason, the tool may be utilized during both the reconnaissance phase and the acquiring access phase.
	Sub finder	Free subdomain detection tool.
	Dmitry	A tool for acquiring information, such as uptime, email addresses, and subdomains. Moreover, the software may be used to accomplish port scanning.
	Burp suite	A sophisticated framework for internet security testing. It may be used to scan for internet flaws automatically, but it also provides innovative guided testing methods to improve each penetration testing step.
Input for PTT	For reconnaissance	Host names, network addresses, IP addresses, and a hardware interface.
	For scanning	Port numbers, IP addresses, network interfaces, and operating system information, among other things, can be found in web URLs.
	For gaining access	OS information, software versions, and protocol versions.

4 $$\:p_{o} \: = 1\: - \prod {\:_{{i\: = 1}}^{n} } \left( {1 - L_{i} } \right)$$

where, n is the number of child nodes of a tree, $\:{L}_{i}$ is the likelihood of occurrence and i is the attack node.

**Time complexity: **The attack defense tree (ADT) consists of attack nodes v, defense nodes d, and intermediate attack node si. To develop a defensive mechanism in the system, the algorithm must traverse the entire ADT. The time complexity of the algorithm is given in Eq. (5).

5 $$\:tc=\:v+i+d$$

**Cost of attack (C**_**o**_**A**_**i**_**): **Assume that the ADT, to be resolved, has attack nodes (nA) and defense nodes (nd), with (nI) inter attack nodes. The algorithm must traverse the whole ADT to develop a defensive mechanism in the system, to obtain the minimum defense cost. The time complexity (tc) of the algorithm is given in Eq. (6). The costs of the attacks in AND (C&) and OR (Co) cases are defined in (7)–(8), respectively^39^.

6 $${\text{tc = nA + nI + nd}}$$

7 $$\:C_{\& } = \:\sum {\:_{{i\: = 1}}^{n} } C_{{A_{i} }}$$

8 $$\:{C}_{o\:=}\frac{\sum\:_{i\:=1}^{n}{L}_{i}\times\:{C}_{{A}_{i}}}{{\sum\:}_{i\:=1}^{n}{L}_{i}}$$

where, Li is the likelihood of occurrences.

**Advantages of attacker (A**_**a**_): The attacker advantage metric with a devastating attack can be determined by using the Eq. (9). $\:{C}_{o}{A}_{i}$variables, according to Maciel R et al., are used to assess the benefit of an attack.

9 $$\:{A}_{a}\:=\:\sum\:_{i\:=1}^{a}{C}_{o}{A}_{i}^{2})$$

**Risk value (r): **The r is obtained by using various techniques like DREAD, and PASTA (attack simulation and threat analysis). Proper risk analysis and evaluation of PASTA methods are used to assess the risk of each detected threat. The main decision in using the PASTA approach is to examine the impact early in the analytical phase rather than address the impact at the risk evaluation stage^50^. In the attack tree, the risk value of each tree node can be determined by using (10).

10 $$\:r\:=\:\left(\left({C}_{r}*{P}_{t}\right)|d\right)$$

where, Cr is the cost value of resource, pt is the probability of threat, and d is the damage of vulnerability. In the DREAD model to compute the level of risks associated with the threats, the risk value can be computed using the parameters: damage, reproducibility (rp), exploitability (ex), discoverability (di) as represented in Eq. (11)^22^.

11 $$\:r=\:\left(d\:+\:{u}_{a}\right)\mathrm{*}\left({r}_{p}\:+\:{e}_{x}\:+\:{d}_{i}\right)$$

**Attack feasibility (F**_*A*_**): **The F_A_ decides the ease of attack for the evaluated scenario. Mathematical equation is given in (12).

12 $$\:{F}_{A}=\:\sqrt[n]{\prod\:_{i\:=1}^{n}{C}_{A}*{N}_{{A}_{i}}*{T}_{{A}_{i}}}$$

where, NAi is the noticeability of attack and TAi is the technical ability of attack.

**Pain factor (f**_**p**_**):** The fp value represents the trouble of the victim if an attack occurs. Evaluation of PF take possible by what level of damage any attack will do to the target system. Equation (13) represents the pain factor.

13 $$\:pf\: = \:\sum {\:_{{i\: = 1}}^{n} } \left( {O_{i} } \right)^{2}$$

where, Oi is the value of operational losses.

**Attack propensity (A**_**p**_**): **Attackers are more likely to carry out attacks that generate a high return with a limited resource expenditure^26^. Attack Propensity is described as the ratio between benefits and costs as mentioned in the Eq. (11).

14 $$\:A_{p} \: = \:\frac{{B_{A} }}{{C_{A} }}$$

where, B_A_ is the attack benefit.

Following^21^, the Ap adopts the combination of feasibility (FA) and attackers advantage (Aa) from attack scenario as mentioned in Eq. (15), attack difficulty (Ad) is given in Eq. (16).

15 $$\:A_{p} \: = \:F_{A} *A_{a}$$

16 $$\:A_{d} \: = \:\frac{1}{{F_{A} }}$$

**Attack frequency or rate of occurrence (A**_**f**_**):** Agiven attack scenario’s propensity (relative frequency) and the number of contacts that take place over that time period determine how often it will occur^51^. The (Af) is given in the Eq. (17). The frequency is referred to as the Annual Rate of Occurrence if the specified time frame is one year. There is a lot of ARO utilized in traditional risk analysis.

17 $$\:{A}_{f}\:=\:{A}_{p}\mathrm{*}{r}_{e}$$

where, re is the rate of encounters.

### Threat tree templates (TTT)

**Fig. 5:**
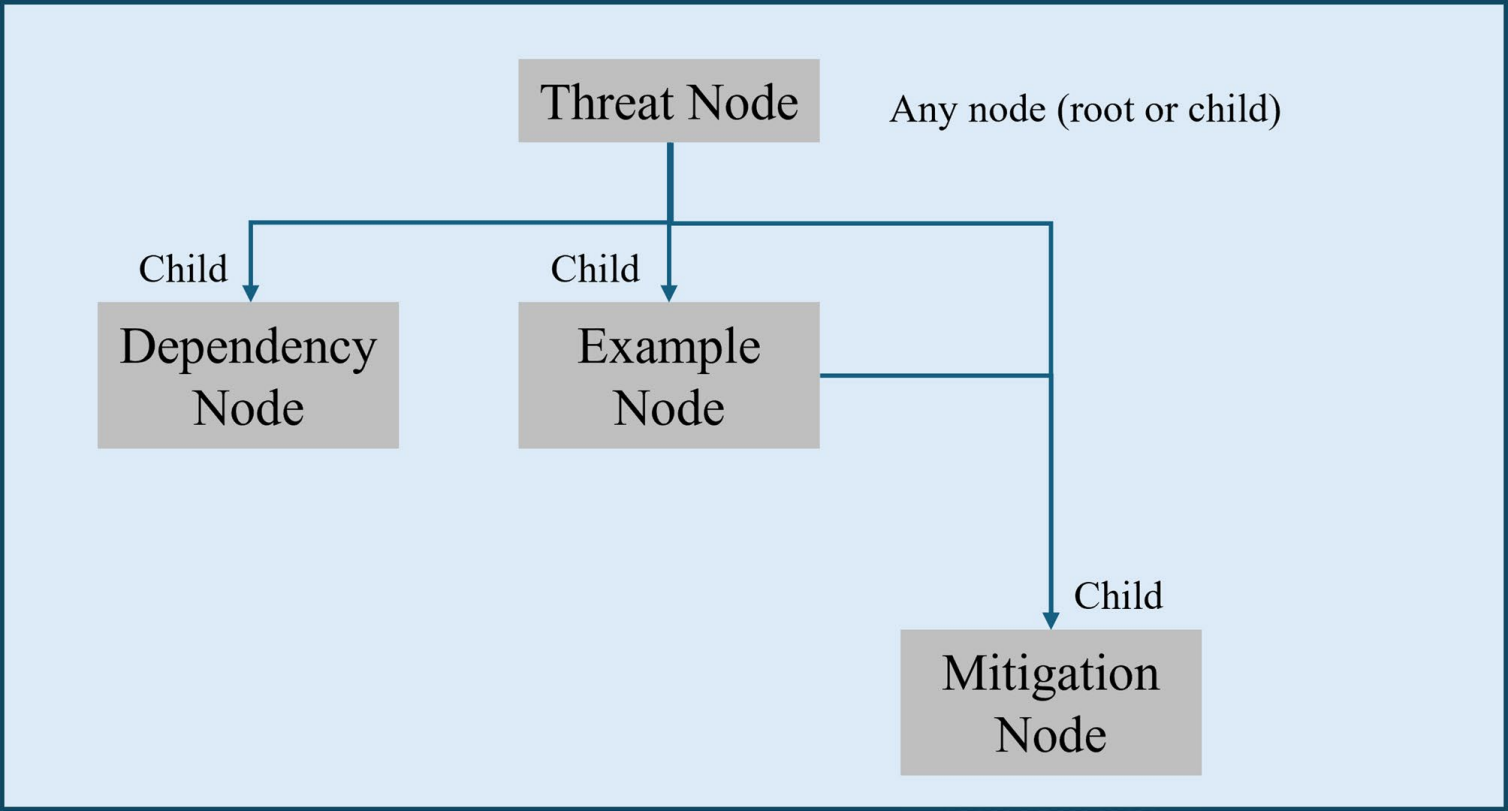
Template nodes of ADT.

A TTT is a collection of duplicate threat trees, completely filled with branches that contain a large number of possible attack schemes, vulnerabilities, and countermeasures. TTT includes actual examples of attack methods, vulnerabilities, and mitigation strategies to help users recognize the threat. This allows analysts to quickly create threat trees for the target sites. The template creators have the proper expertise in security; on the other hand, the users of the template are not security experts. Keywords are created to quickly filter out branches that are unnecessary for the victim machine. A TTT is made up of various components or nodes, such as a threat, dependency, example, or mitigation^23^. Figure 5 illustrates all the types of nodes used for creating a template for the attack tree and attack-defense tree. The threat node can act as any other node. A threat node may be found as a root or child node. The dependency node must be a child of a threat node. The dependency of a node reflects that the parent node can be identified based on another threat tree. An example node portrays an attack or vulnerability for a parallel threat. Mitigation must be a descendant of a threat or an example node, and it serves as a protective measure for its parent node.

## Penetration testing for robotic device

**Fig. 6:**
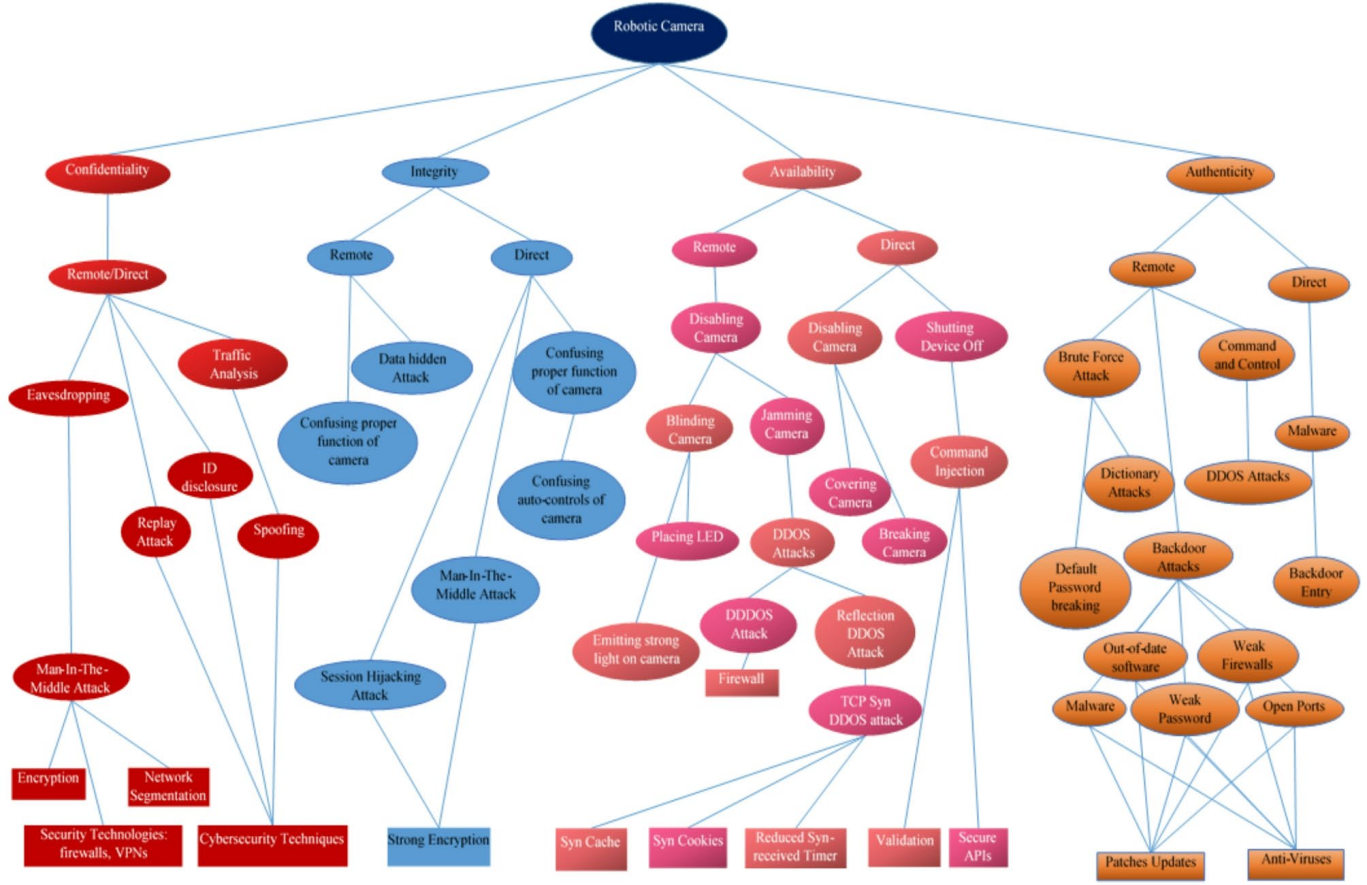
A taxonomy of attacks and mitigations of robotic cameras.

The concept of the Internet of Robotic Things poses new security challenges because of the billions of smart end-devices networked together in wireless networks and connected to the Internet. ADT can be applied in wireless technologies to overcome attacks on data transmission. ADT can be created for fabrication, interception, and domination attacks on Wi-Fi, Bluetooth, ZigBee, etc. ADT can also be applied to IoRT sensors to overcome these attacks^10^. There are various possible attacks on robotic cameras. The figure below shows a taxonomy of the camera attack-defense tree. The proposed taxonomy depicts both the possible attacks as well as the solutions for the robotic attacks (Fig. 6).

A robotic camera, also known as an intelligent camera (sensor), is a machine vision system. In addition to the image-capturing circuitry, robotic cameras can retrieve data specific to an application from collected pictures, as well as provide event descriptions or make decisions for use in automation machines. Robotic cameras play an important role in smooth robotic navigation. There are various possible attacks on cameras; we have proposed some suggestions to defend against the threats or attacks imposed on the IoRT system. The confidentiality of camera data is hindered by various direct or remote attacks such as eavesdropping, man-in-the-middle attacks, traffic analysis, spoofing, replay attacks, wormhole attacks, ID disclosure, etc. To overcome confidentiality attacks, various defense techniques are used, including encryption, network segmentation, firewalls, VPNs, etc. Camera data integrity can be compromised by remote and direct attacks. In remote attacks, data digging attacks are involved and can confuse the proper functioning of the camera. In direct attacks, session hijacking and man-in-the-middle attacks are possible. Additionally, the proper functioning of cameras can be disrupted by tampering with the camera’s auto-controls.The data availability of cameras is vulnerable to remote and direct attacks. Remote attacks include disabling cameras through blinding or jamming them via DDoS attacks. Direct attacks include disabling the camera by physically breaking it or covering it^18,19^. Shutting the camera through a direct attack is possible via command injection. To overcome availability attacks, certain technologies are required, such as firewalls, SYN cache, SYN cookies, reduced SYN received timers, secure APIs, validation, etc. Camera data authenticity is also vulnerable to direct and remote attacks. Remote commands like DoS attacks can compromise authenticity. Direct attacks include brute force attacks, dictionary attacks, and malware. Malware assaults are malicious software that cause damage to a computer, client, server, internet connection, or infrastructure without the awareness of the end user. Most malware types fall into one of the following categories: virus, worm, Trojan horse, adware, spyware, etc., as well as backdoor attacks. Other threats to camera data authenticity include weak passwords, outdated software, and open ports. To defend data authenticity, various defense mechanisms are used, such as antivirus software and patch updates. AT has a corresponding defense mechanism called ADT, as mentioned above—where for each attack, a defense mechanism is present to overcome any catastrophe involving confidential data^6,19–21,41^.

### Penetration testing framework

**Fig. 7:**
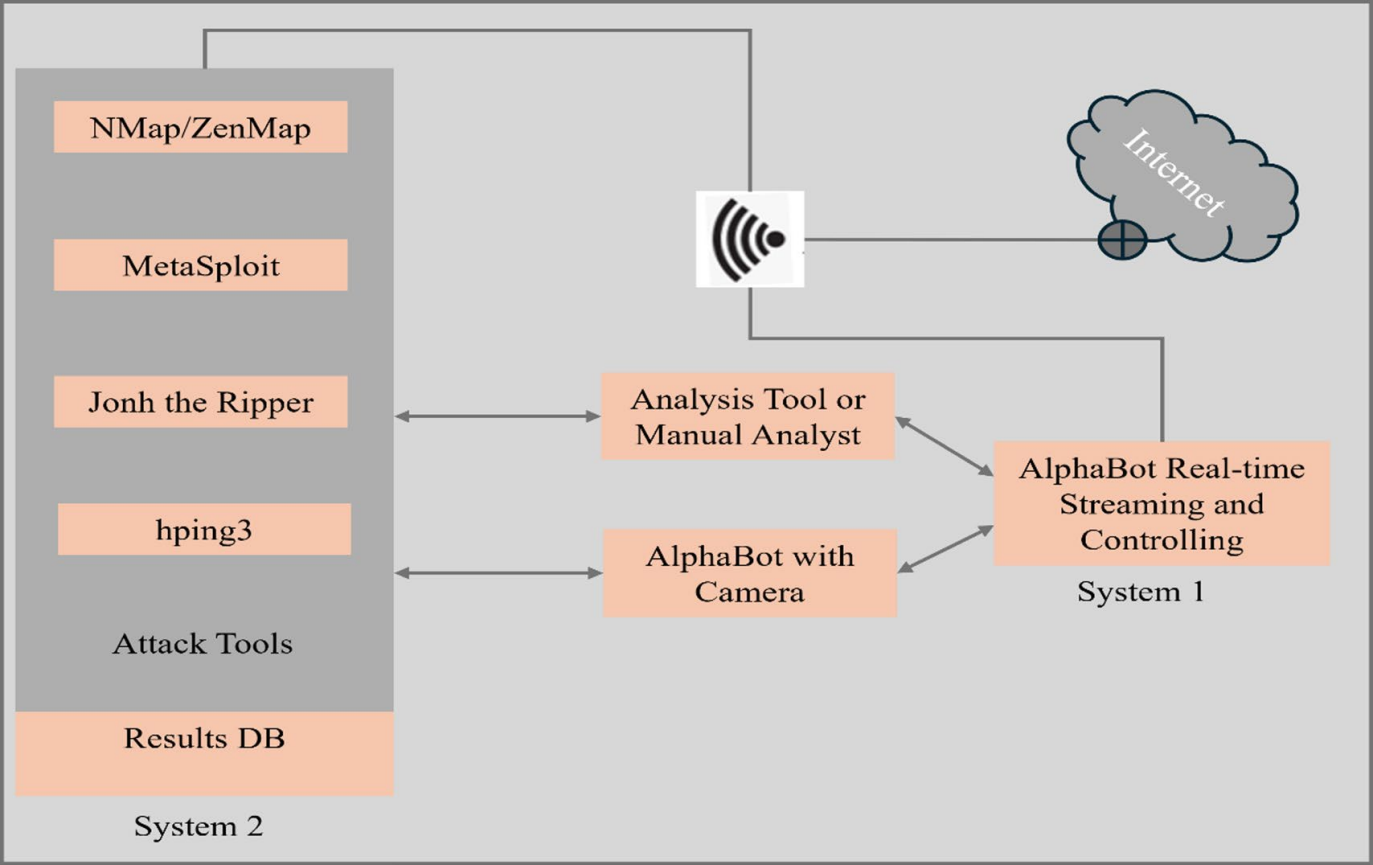
Penetration testing framework.

**Fig. 8:**
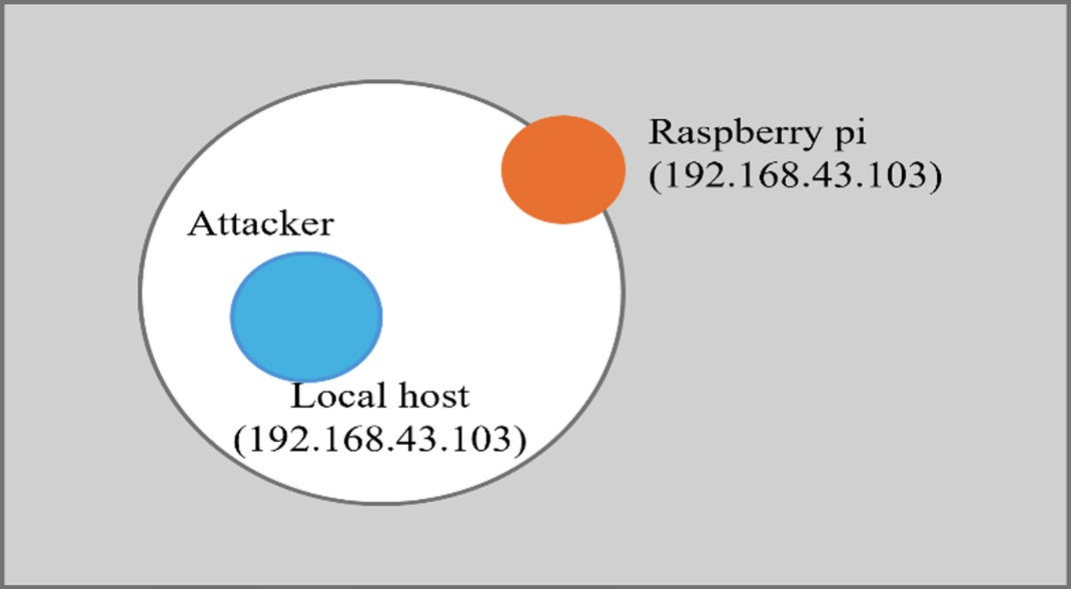
Network topologies of the Raspberry Pi and the adversary.

**Fig. 9:**
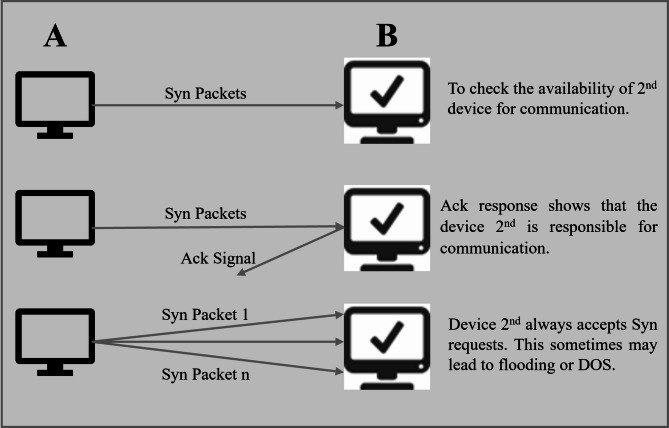
Communication between two devices and the flooding of Syn packets.

The proposed threat model can be used in automatic devices like autonomous cars, humanoid robots, smart surveillance cameras, etc. In this proposed work, out of various possible attacks, we have performed a DoS attack on our robotic device using penetration testing methods. Figure 7 depicts the penetration testing technique. In this procedure, we have two systems: System 1 and System 2. System 1 handles control and real-time streaming, while System 2 handles attack tools and a database for penetration testing outcomes. Once an AlphaBot with a camera is connected to the internet, real-time streaming between AlphaBot and System 1 takes place. The following penetration attack tools are utilized to construct assaults against the AlphaBot camera: Nmap/ZenMap, Metasploit, John the Ripper, hping3, and others. During this phase, we performed flooding attacks on AlphaBot using the attack tools. The attack tools responsible for the DoS attack are Metasploit and hping3. Nmap/ZenMap handles scanning and reconnaissance, whereas John the Ripper handles password attacks.In our penetration testing environment, we had various connected tools like the Kali Linux machine, AlphaBot, and computer systems. The computer system is installed with the Kali Linux operating system. ZenMap, a pen-testing tool available in Kali OS, is a GUI version of Nmap. A proper connection is established among the various components. AlphaBot with Raspberry Pi acts as a computational model for AlphaBot. The Raspberry Pi is considered the minicomputer built inside AlphaBot and provides capabilities such as capturing, sending signals, and navigation. Computer systems and AlphaBot should be on the same network to obtain IP addresses of the same class for local accessibility. This means a private IP address with the same network access is required, and no external entity can access it. Computers and AlphaBot connect using a direct connection via network topologies. Open ports (scanned through ZenMap and Metasploit), such as 22 and 5900, are used. The database stores the results of each operation. Figure 8 represents the network topologies for Raspberry Pi; these topologies are used for generating exploit traffic. The attacker system running Metasploit and the target system (AlphaBot) are placed in the same network. Figure 9 illustrates the communication process between two nodes, say A and B. SYN packets are sent from A to B to check the availability of device B. When device B responds with an ACK signal, communication is possible. In this way, the device is responsible for the communication process. Device B always accepts SYN requests, but this behavior makes it vulnerable to attacks. For example, if there are 10,000 SYN requests sent simultaneously to B, it must respond to all. But this is not feasible. It acts as a flood on device B. In this way, device B becomes computationally weak and less functional. Thus, a flooding or DoS attack is possible^9^.

### Penetration testing working

**Fig. 10:**
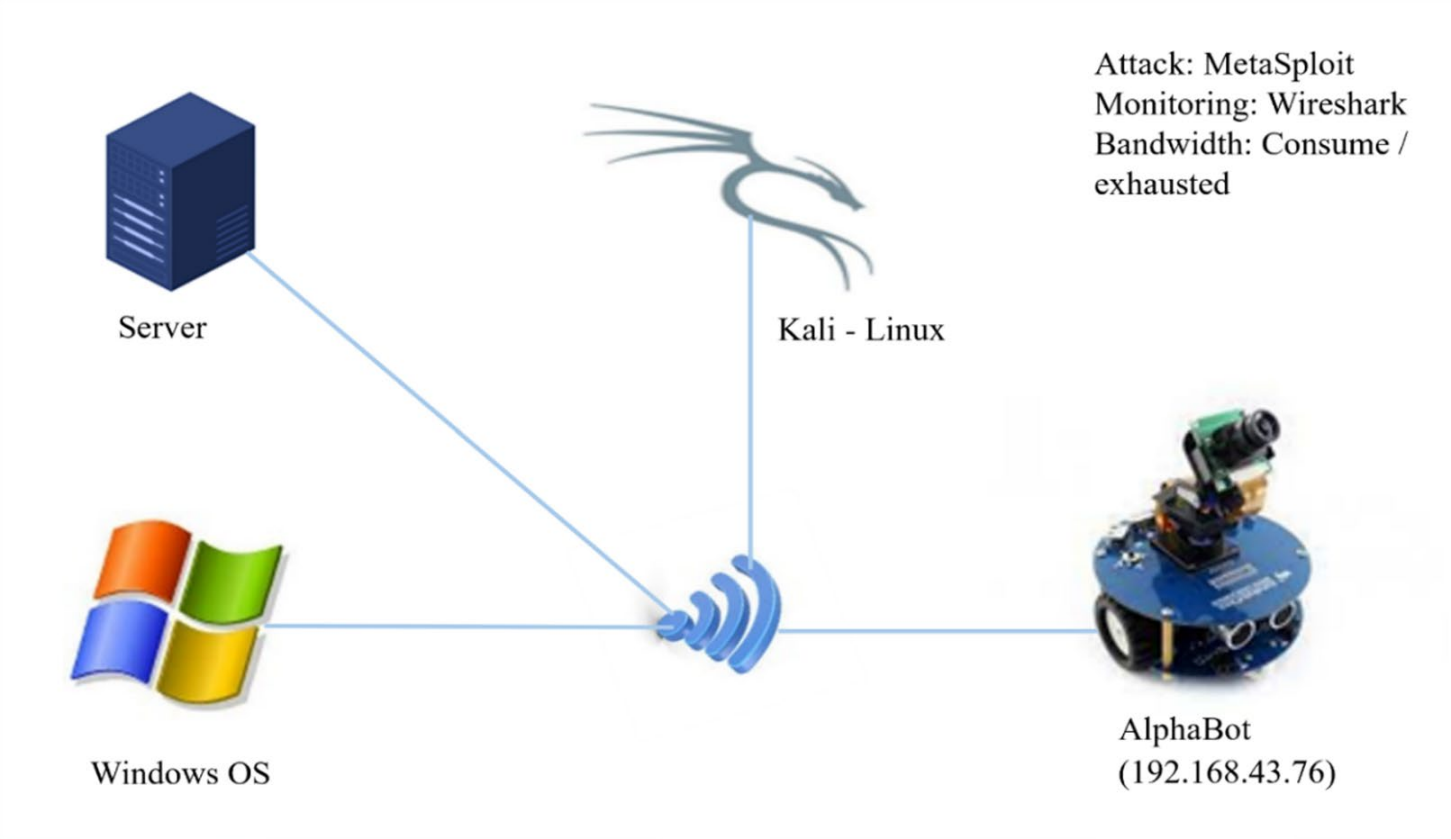
The proposed penetration testing framework.

The testing framework is comprised of an attacker machine—Kali Linux, AlphaBot, Windows OS, and a server—and all of these systems are networked via an internet connection. Metasploit consists of auxiliary files, such as basic-related, scanning (SYN and port scanning), encoding, etc. Metasploit exploits vulnerabilities and injects payloads (viruses) into the system. Kali Linux serves as the attack machine. An attack can be launched on AlphaBot from Kali Linux. Wireshark can be used to monitor performance on the same or different systems; performance monitoring on Windows can be done, but the network must be the same. An image-based penetration testing framework is proposed in Fig. 10. For connectivity, we used a Wi-Fi hotspot, and all devices were connected to it.

**Scanning: **First, using Kali Linux, ZenMap performs scanning to check which ports are open on the target IP address. That is, port scanning is done using ZenMap in Kali Linux, and ports 22 and 5900 are found to be open. Then, the operating system in use is identified, and its performance is also assessed. We then retrieve the MAC address of AlphaBot and the operating system it is running. This forms the basis of scanning and reconnaissance.

**Fig. 11:**
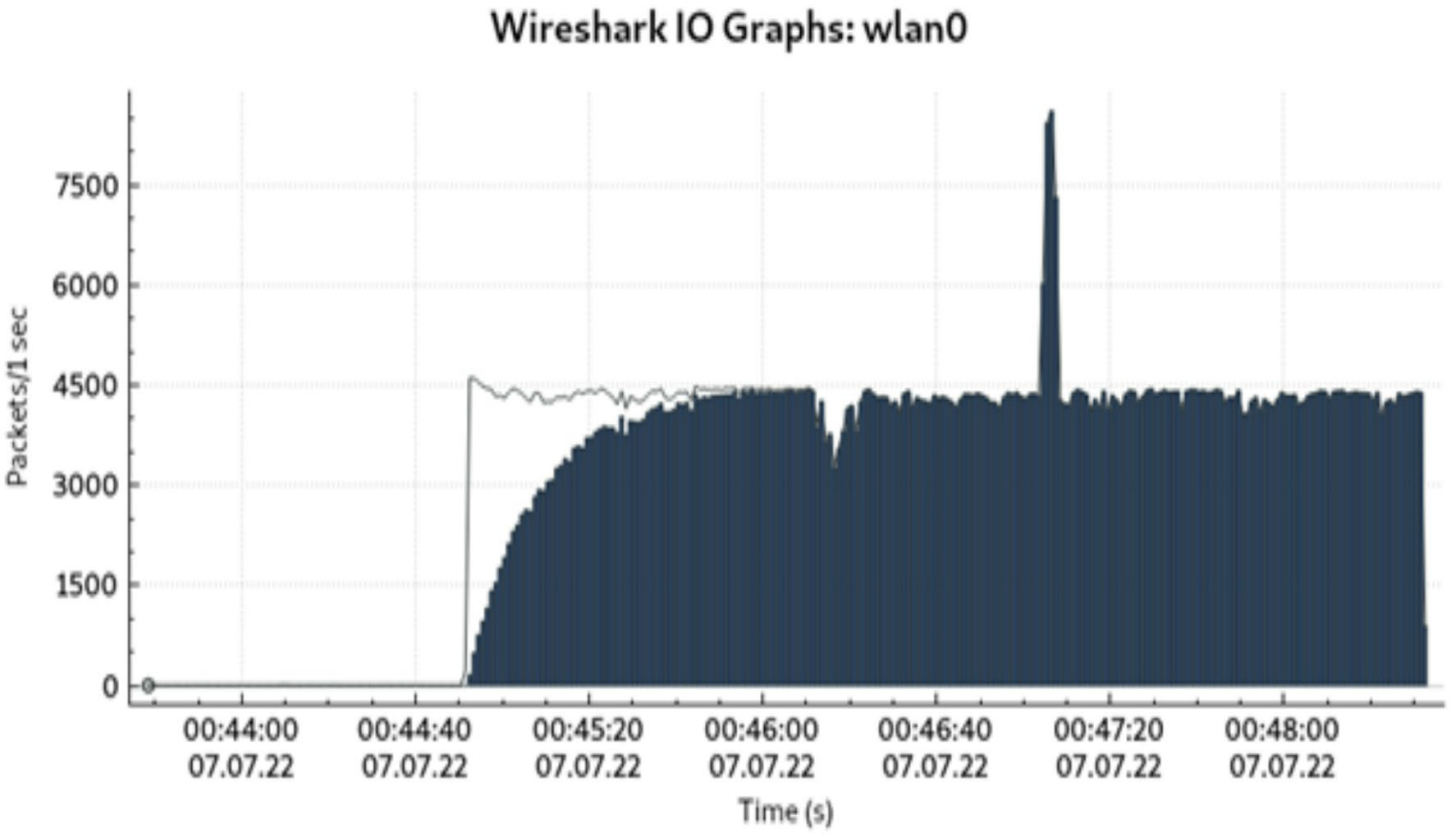
A representation of the traffic flow during the DoS attack.

**DoS attack: **Before the DoS attack, no data flow was occurring in communication. After the DoS attack, all requests become visible, and all packets can be monitored, as shown in Fig. 11. For the DoS attack mechanism, the Metasploit framework is used. In Metasploit, auxiliary packages are required for performing the SYN flood mechanism. This involves flooding the target with SYN packets. All Metasploit activities are performed on Kali Linux by the attacker. For transmission of data from device A to device B, we need the IP address of B; many attributes are shown here, such as time and host, as presented in Table 9. We have configured a large timeout so that continuous flooding can be sustained. ZenMap runs a system scan to obtain the desired information. Then, after selecting the appropriate auxiliary files, the IP address, port number, and timer are configured. The attack is then carried out, the system’s overall performance is monitored, and the results are collected. Finally, the program is terminated. The SYN request flooding mechanism commands from the attacker’s file in Linux are given below.

- Use MSFconsole.
- Use auxiliary/dos/tcp/synflood.
- Show options.
- Set RHOSTS 192.168.43.76.
- Set RPORT 22.
- St Timeout 9,999,999.

**Table 9:** A summary of system scanning.

Raspberry Pi (192.168.43.76)		
Host status	State	Up
	Open ports	2 (22, 5099)
	Filtered ports	0
	Closed ports	998
	Scanned ports	1000
	Uptime	2,449,446
	Last boot	Tue Jun 716:54:48 2022
Addresses	IPV4	192.168.43.76
	IPV6	Not available
	MAC	B8:27:EB:88:72:9 A
Host names	Name-Type	Raspberry Pi PTR
Operating system	Name	Linux 2.6.32
	Accuracy	96%
Ports used	Port-protocol-state	22-tcp-open
	Port-protocol-state	1-tcp-closed
	Port-protocol-state	32,497-udp-closed
New distance	1 hop	New distance
Uptime id chosen		

## Results and discussions

Considering the importance of security in robotic systems, we discussed some important threat modeling and penetration testing methods. The threat modeling methods ensure the identification and evaluation of possible threats prior to the actual development of the robotic system, whereas penetration testing provides a mechanism to evaluate the level of security in a deployed or developed robotic system. Both pre- and post-development security assessment methods are equally important to ensure the security of robotic systems from known as well as zero-day attacks. With this consideration, we have examined an AlphaBot robotic device in general and its camera component in particular to identify the possible threats associated with it and, accordingly, formulate an attack tree and an attack-defense tree. The attack tree defines the possible threats that can exploit the security of the robotic camera. Here, the ultimate aim of the attacker is to damage the camera device, and there exist many alternate paths for an attacker to achieve this goal. The goal of the attacker is represented as the root node of the attack tree, and the intermediate nodes define the paths to reach the goal node. To begin with, the attacker exploits the threats defined as the leaf nodes of an attack tree and, through the intermediate nodes, reaches the root node. Based on the threats associated with a robotic camera device in the existing reviewed literature, we formulate a complete attack tree and an attack-defense tree. An attack-defense tree prevents the exploitation of threats defined in the leaf nodes. By preventing them, the attacker is deprived of access to the intermediate nodes and, thereby, the root node. This ensures complete protection of the robotic device. Moreover, we discuss the use of threat models for threat identification (such as STRIDE) and risk evaluation (such as DREAD and PASTA). The risk evaluation mechanism is used to assess the risk associated with the identified threats. Based on the level of associated risk, the most severe threats can be prioritized for early mitigation.

**Fig. 12:**
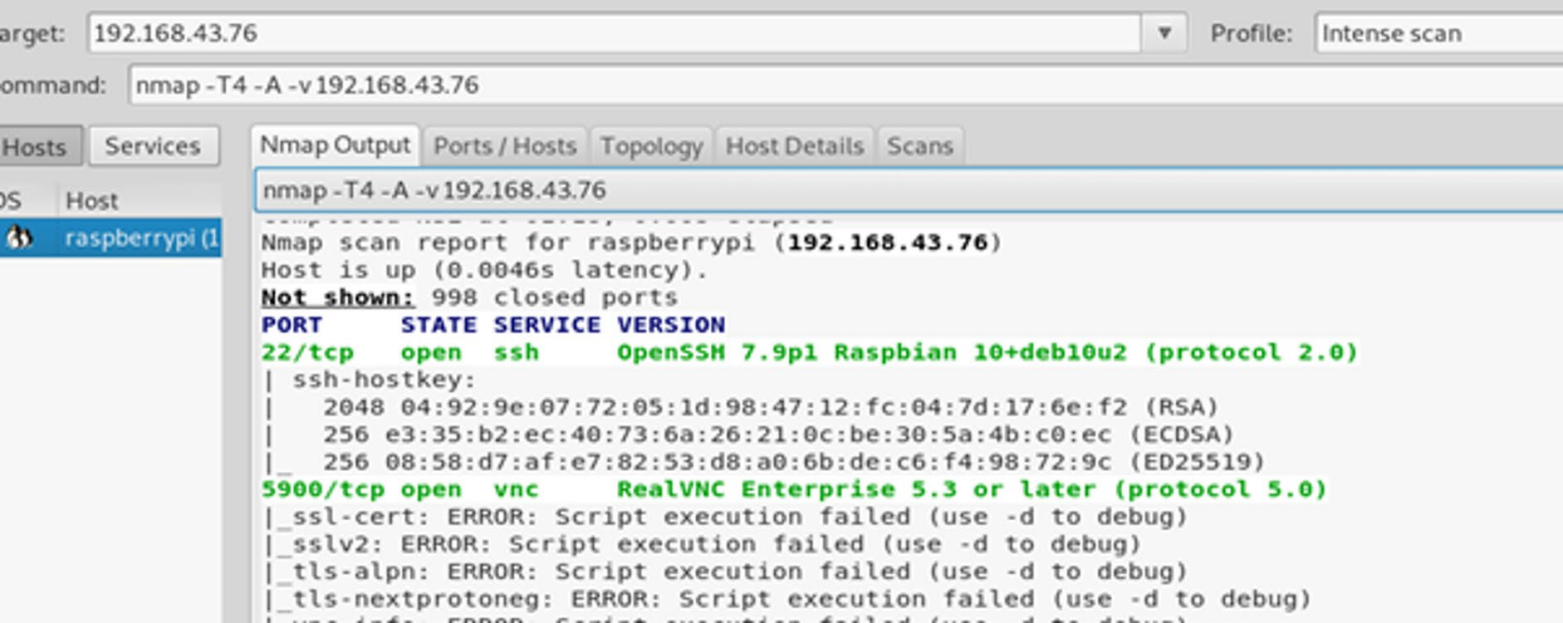
AlphaBotreconnaissance through NMap scanning.

The penetration testing aspect of the security assessment and evaluation mechanism covers developed robotic systems, wherein the system can be tested for security loopholes. In our methodology, we considered an AlphaBot robotic device for penetration testing. We performed reconnaissance through Nmap to obtain device credentials such as device name, MAC address, firmware/OS, open ports, etc., as shown in Fig. 12. Based on the information obtained, we identified that port number 22 is open on the device and is running an SSH service. This means the port is actively listening and waiting for incoming packets. This could be dangerous for robotic devices if proper filtering of incoming packets is not implemented. An attack in the form of Denial of Service (DoS) could compromise the availability of the entire system solely due to an unmanaged open port.

**Fig. 13:**
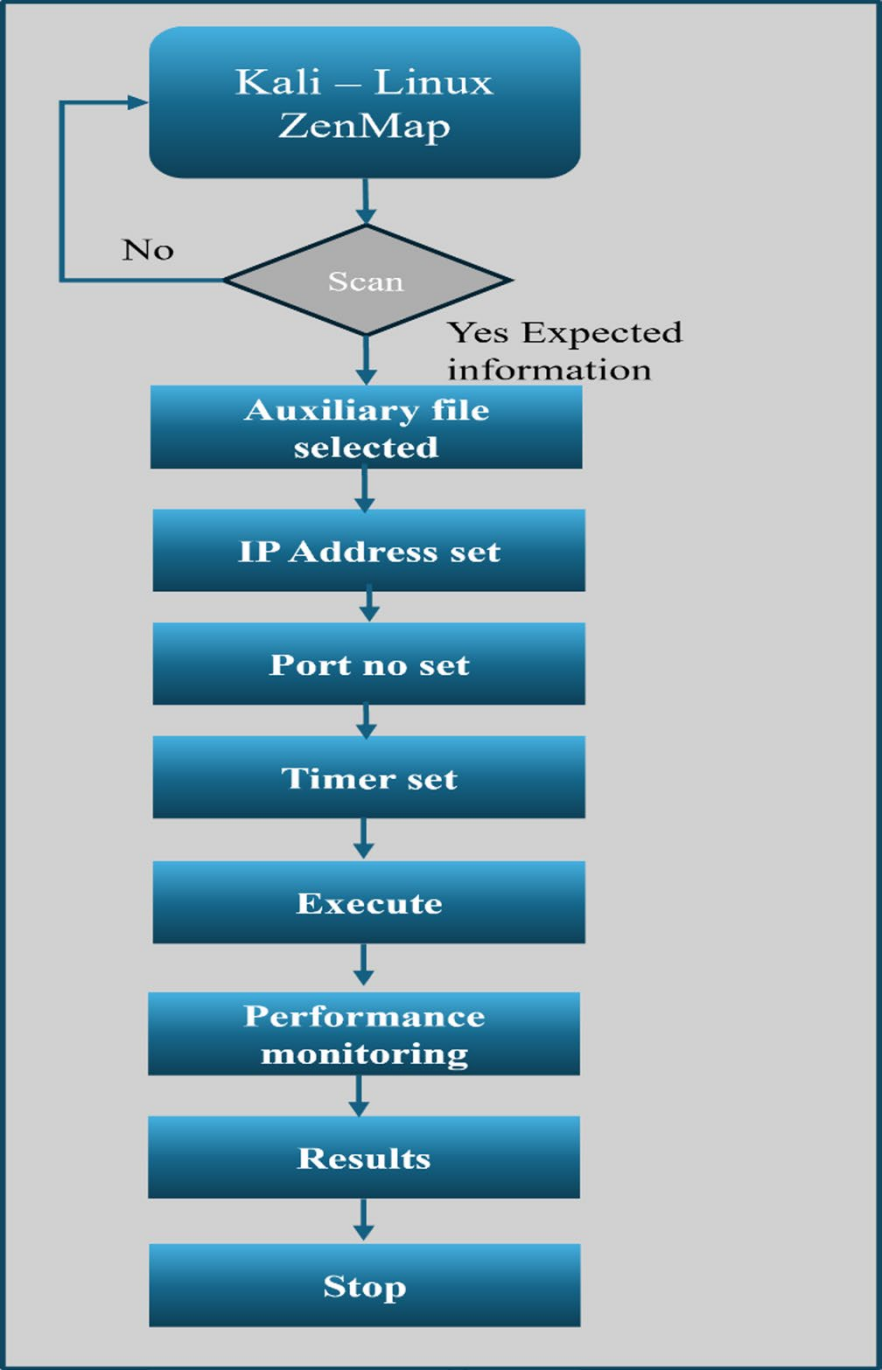
Penetration testing flowchart.

**Fig. 14:**
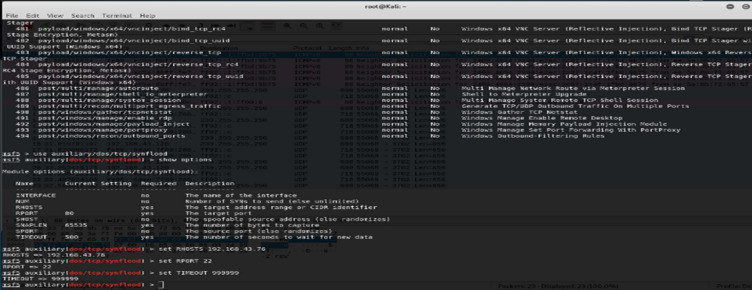
DoS attack on alphabot through the metasploit.

Under the proposed framework methodology (Fig. 13), a DoS attack is performed on the AlphaBot robotic device using Metasploit (Fig. 14).The attacker running Metasploit has the IP address 247.118.35.109, and the victim’s IP address is 192.168.43.76. We performed flooding of SYN packets on port number 22, as shown in Fig. 14.

**Fig. 15:**
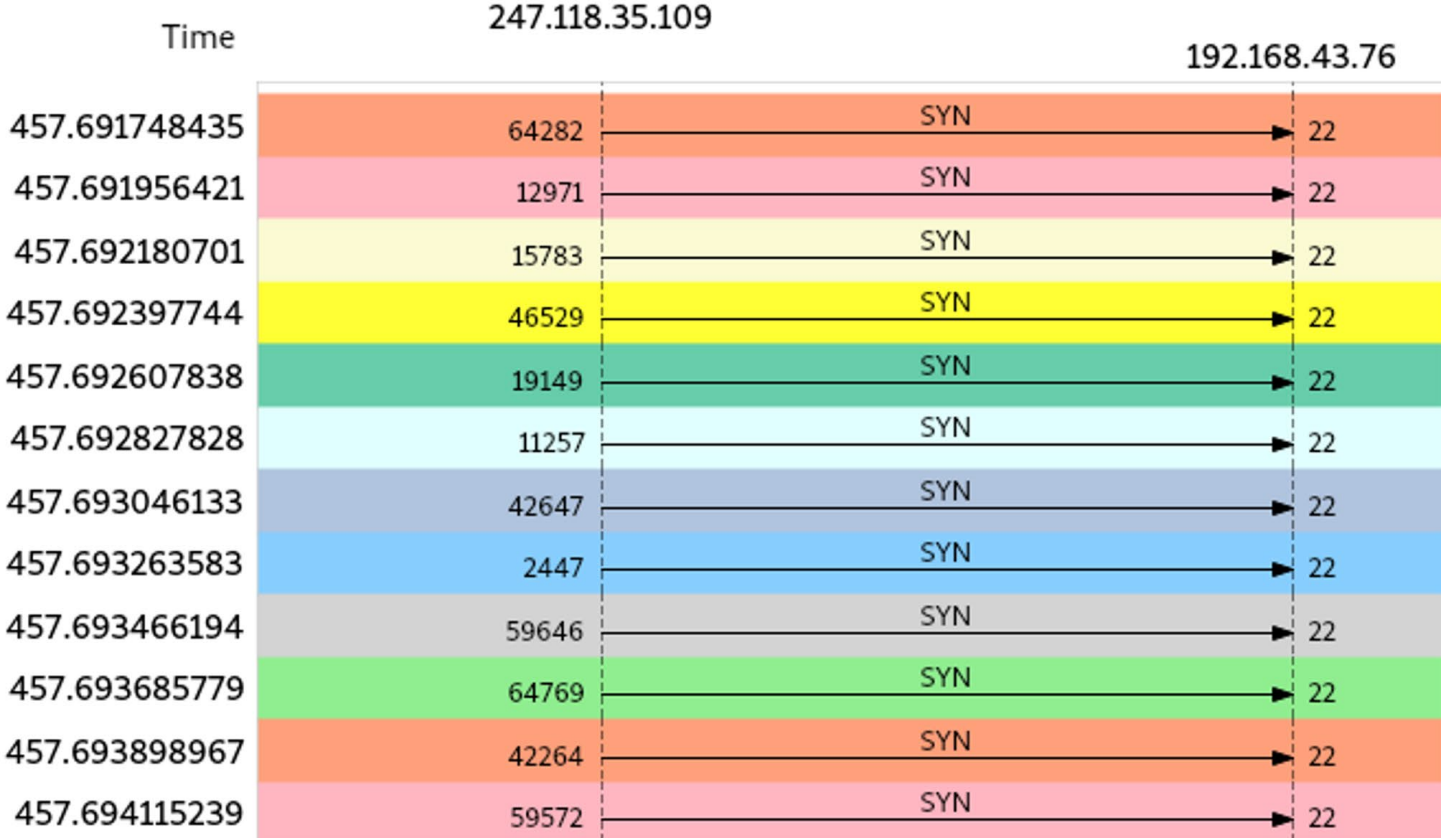
A continuous transmission of SYN packet towards the victim (AlphaBot).

In Metasploit, we searched for DoS and flooding exploits and auxiliaries, and we found a SYN flooding-based module. We selected the module with the use command. After selecting the module, we provided the victim’s IP address and port number using the commands set RHOSTS 192.168.43.76 and set RPORT 22, respectively. Moreover, we invoked another command set TIMEOUT 9,999,999 to continue flooding for 9999 s, or approximately 166 min. The aim of using such a large timeout value was to perform continuous flooding on the AlphaBot. Once we prepared our module, we used the run or execute command, which in turn initiated the flooding on the AlphaBot device with IP address 192.168.43.76. It can be clearly seen from Fig. 15 that Metasploit (attacker) continuously sent SYN packets with different sequence numbers toward the AlphaBot (victim). Also, as observed from Fig. 11, initially there was no traffic flowing toward the AlphaBot, but upon invocation of the DoS attack, an average of 4500 packets per second began flowing toward the AlphaBot. The traffic flow depicted was captured using the Wireshark tool. The exponential rise in traffic on the AlphaBot device can render the entire system unresponsive to various services. For instance, a robotic device intended to capture real-time streaming through its camera component may have its real-time communication with a base station disrupted, making such communication unavailable due to the DoS attack. Therefore, there is a need to consider effective management of open ports and filtering of incoming packets in robotic devices.

**Fig. 16:**
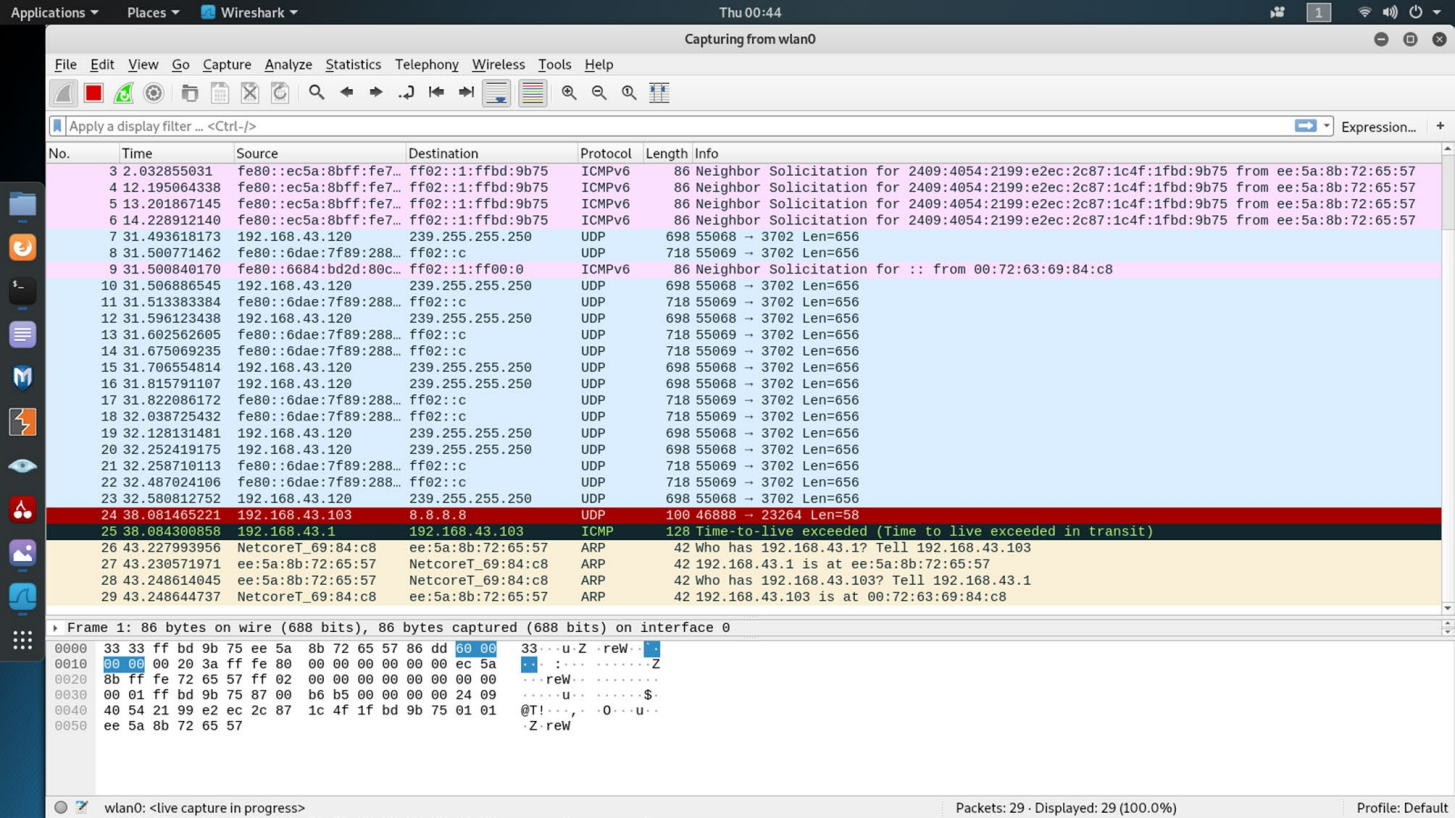
Inspection of the captured packet during the DoS attack.

**Fig. 17:**
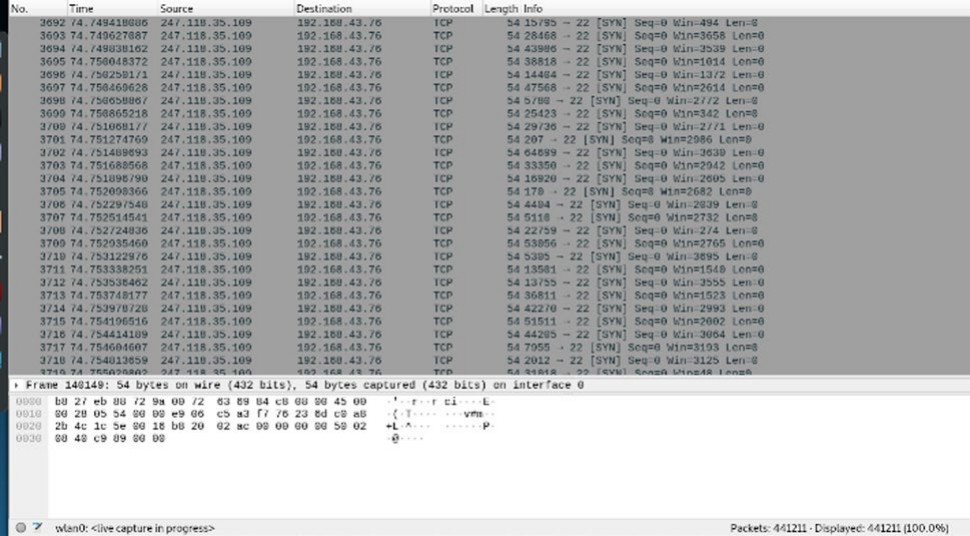
Flooding by the attacker (247.118.35.109) on the receiver (192.168.43.76).

Then, Wireshark, as shown in Fig. 16, monitors the attack procedure. Here, Wireshark checks the source and destination IP addresses of the attacker and the victim, and also verifies the protocols being used. Flooding by the attacker (247.118.35.109) on the receiver (192.168.43.76) takes place, as shown in Fig. 17.

### Limitations and future scope

While this study establishes a comprehensive framework for IoRT security assessment, certain limitations must be acknowledged. First, the experimental validation is confined to a single robotic platform (the AlphaBot). While the AlphaBot serves as a highly representative model for resource-constrained edge robotic devices^50^, future research should adapt this framework across a heterogeneous swarm of robotic architectures (e.g., UAVs^12^ or industrial manipulators). Second, the penetration testing scope in this work intentionally prioritizes availability threats, specifically demonstrating SYN flooding-based DoS attacks. Future iterations of this research will expand the Attack-Defense Tree (ADT) and testing parameters to encompass integrity and confidentiality breaches, such as sensor spoofing, command injection, and physical layer attacks^5^.

## Conclusion

The field of IoRT is characterized by the necessity for substantial data transmission between robotic entities, cloud storage, and various interconnected devices. However, this extensive data transmission also introduces vulnerabilities, leading to potential data breaches and cyberattacks, thereby escalating the gravity of security concerns. Consequently, it is imperative to address these security challenges. To ensure secure data transmission within IoRT systems, the implementation of secure and trusted data-sharing mechanisms is recommended, aiming to mitigate existing research gaps. This study underscores the critical role of threat modeling and penetration testing methodologies in the assessment of security risks. The proactive use of these methods, both in the pre-design phase and post-development, is essential to guarantee the security of robotic devices. Notably, threat modeling serves as the foundation for security assessments. This paper explores contemporary threat models, including STRIDE, DREAD, PASTA, Attack Tree, and Attack Defense Tree. These models are instrumental in identifying potential threats and assessing associated risks, considering various attributes such as the likelihood of attack occurrence, time complexity, cost, and frequency of attacks. The study extends its focus to the security of robotic cameras, devising specific attack tree and attack defense tree models to visualize potential threats and corresponding countermeasures. Additionally, penetration testing is employed to evaluate system vulnerabilities, utilizing tools such as Nmap and Metasploit to extract critical device information, including MAC addresses, open ports, and operating systems. This information can be exploited to initiate Denial-of-Service (DoS) or flooding attacks. The study’s experimentation demonstrated noticeable changes in network traffic patterns on the AlphaBot robot before and after the DoS attack was invoked. It is worth noting that mismanaging open ports and neglecting incoming packet filtering can pose serious risks to critical robotic applications, such as driverless cars. To mitigate potential human and financial losses, future IoRT systems must prioritize enhanced security measures. Consequently, future technological advancements should consider the proposed security framework and the trusted data-sharing mechanism outlined in this study. The envisaged threat model can find valuable applications in a wide range of autonomous devices, such as autonomous cars, humanoid robots, and smart surveillance cameras, contributing to a more secure and resilient IoRT landscape.

## Data Availability

The datasets generated and analysed during the current study are available in the GitHub repository Warrafeeq/IoRT-Security-Research at [https://github.com/Warrafeeq/IoRT-Security-Research](https:/github.com/Warrafeeq/IoRT-Security-Research) .
